# Cryo-EM structure of the inner ring from the *Xenopus laevis* nuclear pore complex

**DOI:** 10.1038/s41422-022-00633-x

**Published:** 2022-03-18

**Authors:** Gaoxingyu Huang, Xiechao Zhan, Chao Zeng, Ke Liang, Xuechen Zhu, Yanyu Zhao, Pan Wang, Qifan Wang, Qiang Zhou, Qinghua Tao, Minhao Liu, Jianlin Lei, Chuangye Yan, Yigong Shi

**Affiliations:** 1 Westlake Laboratory of Life Sciences and Biomedicine, Hangzhou, Zhejiang China; 2 Key Laboratory of Structural Biology of Zhejiang Province, School of Life Sciences, Westlake University, Hangzhou, Zhejiang China; 3 Institute of Biology, Westlake Institute for Advanced Study, 18 Shilongshan Road, Hangzhou, Zhejiang China; 4 Beijing Advanced Innovation Center for Structural Biology & Frontier Research Center for Biological Structure, Beijing, China; 5 Tsinghua University-Peking University Joint Center for Life Sciences; School of Life Sciences, Tsinghua University, Beijing, China

**Keywords:** Cryoelectron microscopy, Nuclear organization

## Abstract

Nuclear pore complex (NPC) mediates nucleocytoplasmic shuttling. Here we present single-particle cryo-electron microscopy structure of the inner ring (IR) subunit from the *Xenopus laevis* NPC at an average resolution of 4.2 Å. A homo-dimer of Nup205 resides at the center of the IR subunit, flanked by two molecules of Nup188. Four molecules of Nup93 each places an extended helix into the axial groove of Nup205 or Nup188, together constituting the central scaffold. The channel nucleoporin hetero-trimer of Nup62/58/54 is anchored on the central scaffold. Six Nup155 molecules interact with the central scaffold and together with the NDC1–ALADIN hetero-dimers anchor the IR subunit to the nuclear envelope and to outer rings. The scarce inter-subunit contacts may allow sufficient latitude in conformation and diameter of the IR. Our structure reveals the molecular basis for the IR subunit assembly of a vertebrate NPC.

## Introduction

Nuclear pore complex (NPC) is present in the nuclear envelope (NE) of all eukaryotic cells and mediates nucleocytoplasmic transport.^1,2^ A vertebrate NPC consists of four circular scaffolds: a cytoplasmic ring (CR), an inner ring (IR), a nuclear ring (NR), and a luminal ring (LR).^3–5^ IR resides in the central layer of the NPC and bridges the outer rings CR and NR. Of the four rings, only the LR resides in the lumen of the NE. The cytoplasmic filaments (CF) are connected to the CR and facilitate nucleocytoplasmic transport. Structural investigation of the NPC has yielded a wealth of information on individual nucleoporins, subcomplexes, and ring scaffolds of the NPC.^2,4,6,7^

A vertebrate NPC has an extraordinary molecular mass and displays considerable conformational flexibility despite its 8-fold symmetry. These aspects have made cryo-electron microscopy (cryo-EM) analysis of the NPC through single particle analysis (SPA) technically challenging. Consequently, cryo-electron tomography (cryo-ET) through sub-tomogram averaging (STA) has been the dominant approach in the structural investigation of NPC during the past two decades. The best resolution achieved for cryo-ET reconstruction of the IR subunit is ~21 Å for the human NPC.^8^ This EM map allowed generation of composite coordinates for human NPC through docking of known X-ray structures.^8^

We took the cryo-EM SPA approach and succeeded in the reconstruction of the CR subunit from the *Xenopus laevis* (*X. laevis*) NPC with a local resolution of ~5–8 Å.^9^ More recently, relying on enhanced methods of sample preparation and cryo-EM analysis, we have markedly improved the local resolution of the *X. laevis* CR subunit to 3.8 Å.^10^ Detailed features of secondary structural elements and bulky amino acid side chains are identifiable in a portion of the core region.

In this study, we report the cryo-EM reconstruction of the IR subunit of the *X. laevis* NPC at an average resolution of 4.2 Å, with local resolution reaching 3.8 Å. The EM maps allow docking of known and predicted structures of *X. laevis* nucleoporins^10,11^ as well as identification of secondary structural elements. Based on the EM maps, we have generated atomic coordinates for 30 nucleoporins of the *X. laevis* IR subunit, which include 19,315 amino acids. This structure reveals the underpinnings of vertebrate IR subunit assembly.

## Results

### Cryo-EM analysis of the *X. laevis* IR subunit

**Fig. 1:**
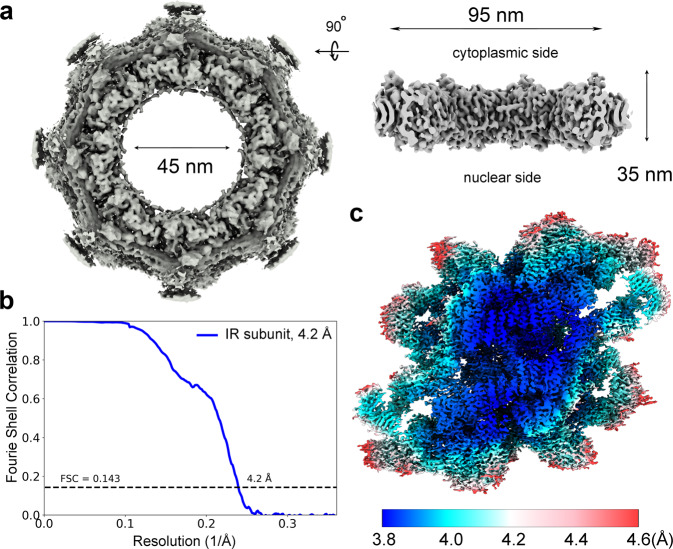
Cryo-EM structure of the IR of the *X. laevis* NPC. **a** Cryo-EM reconstruction of the IR of *X. laevis* NPC at 22 Å resolution. Two perpendicular views are shown. The inner diameter of the IR is ~45 nm. **b** The final EM reconstruction for the IR subunit displays an average resolution of 4.2 Å. Shown here is a curve of the Fourier Shell Correlation (FSC) over resolution. **c** Distribution of the local resolution for the EM reconstruction of the IR subunit. The color-coded resolution bar is shown below.

The same cryo-EM datasets used for reconstruction of the CR subunit from *X. laevis* oocytes^10^ were used for reconstruction of the IR subunit. A total of 800,825 NPC particles were manually selected from 33,747 micrographs (Supplementary information, Fig. S1a). With C8 symmetry, the intact IR was reconstructed at an initial resolution of 22 Å using 660,302 NPC particles (Fig. 1a; Supplementary information, Fig. S1a, b), allowing extraction of the IR subunits (Supplementary information, Fig. S2a). Using data with the pixel size of 2.774 Å, we generated a reconstruction of the IR subunit at 5.6 Å resolution. The refined subunits were projected back to the original IR, followed by subunit re-extraction (Supplementary information, Fig. S2b) with a pixel size of 1.387 Å. Eventually, 2,093,631 particles yielded a 3D EM reconstruction of the IR subunit at an average resolution of 4.2 Å, with local resolutions reaching 3.8 Å (Fig. 1b, c; Supplementary information, Figs. S3–S8 and Tables S1, S2).

Compared to previous studies,^8,12^ the improved EM maps allowed unambiguous assignment of most components in the IR subunit and accurate placement of secondary structural elements. We identified 30 molecules of nine distinct nucleoporins in each IR subunit, including four copies of Nup93, six copies of Nup155, four copies of channel nucleoporin hetero-trimer (CNT, each comprising Nup62/Nup58/Nup54),^13^ and two copies each for Nup205, Nup188, NDC1, and ALADIN.

The atomic coordinates of Nup93 and Nup205 from the CR subunit^10^ were docked into the EM map with little adjustment. Using AlphaFold,^11^ we generated atomic coordinates for each of the other *X. laevis* nucleoporins. We individually docked each predicted structure into the 4.2 Å EM map and made minor adjustment (Supplementary information, Figs. S4–S8). Compared to the core region, the EM density in the peripheral region of the IR subunit has a slightly lower resolution (Fig. 1c; Supplementary information, Fig. S7). Nonetheless, the AlphaFold-predicted structure of the ALADIN–NDC1 hetero-dimer fits the EM density reasonably well. The final molecular model of the *X. laevis* IR subunit contains 18,742 amino acids, with 772 α-helices and 296 β-strands (Supplementary information, Figs. S9–S14 and Table S3).

### Overall structure of the IR subunit

**Fig. 2:**
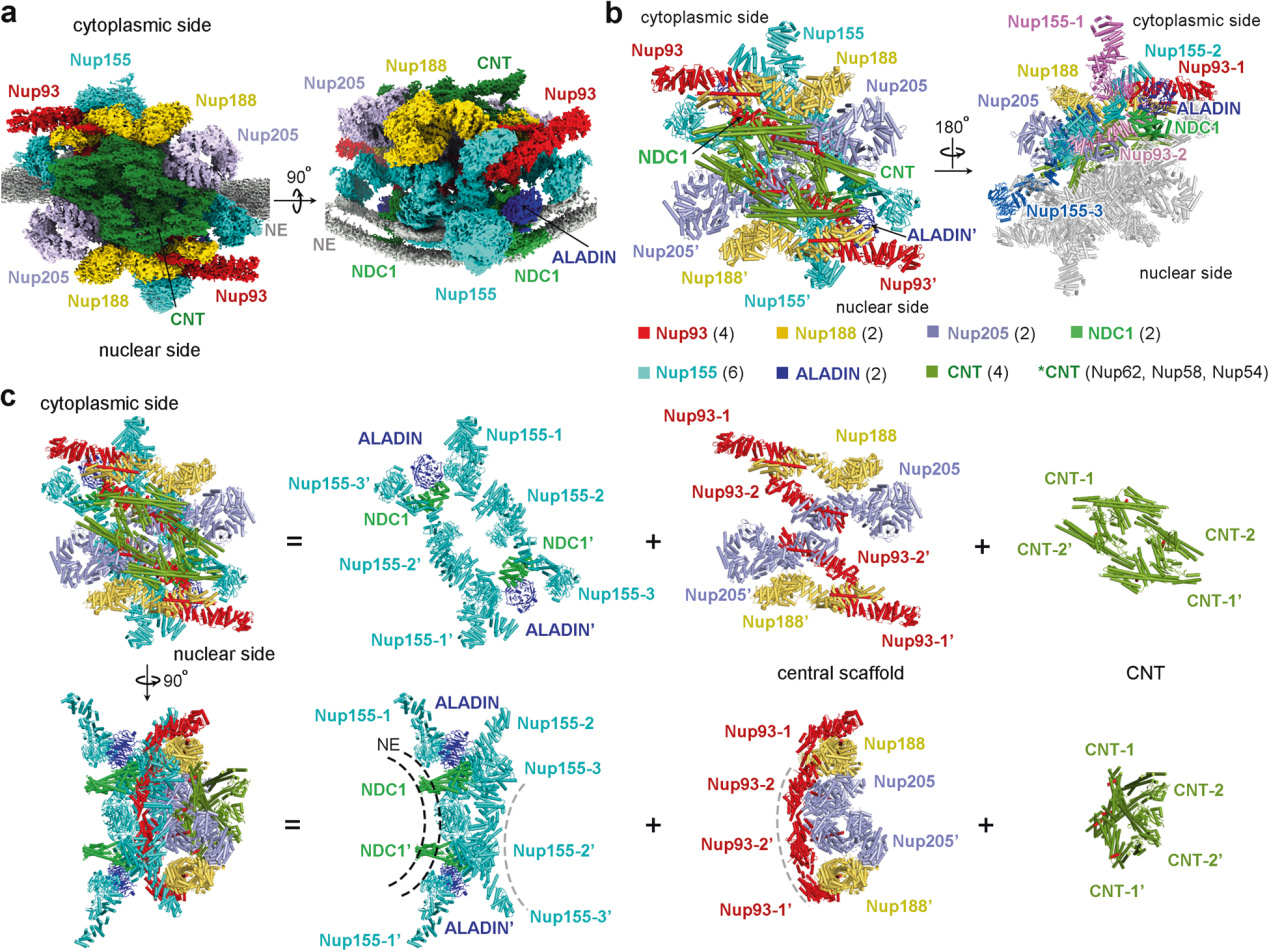
Overall structure of the IR subunit from the *X. laevis* NPC. **a** Overall EM map of the IR subunit from *X. laevis* NPC. Two perpendicular views are shown. The IR subunit displays a 2-fold symmetry, with a cytoplasmic half and a nuclear half. The EM maps for individual nucleoporins in each half are color-coded identically. The NE is shown in gray, the two layer-like density representing lipid bilayer of NE is apparent in the right panel. **b** Structure of the IR subunit from the *X. laevis* NPC. Two views are shown. In the left panel, individual nucleoporins are color-coded. In the right panel, nucleoporins of the nuclear half are colored gray; different copies of the same protein in the cytoplasmic half are differentially colored. **c** Overall structure of the IR subunit from *X. laevis* NPC. Top and bottom: the overall structure of the IR subunit is displayed in two perpendicular views (first column from left). For each view, the IR subunit is disseminated into three layers: ten molecules of Nup155/ALADIN/NDC1 close to the NE (second column from left), the central scaffold of eight molecules of Nup188/Nup205/Nup93 (third column from left), and four CNTs (fourth column from left).

The IR subunit displays a 2-fold symmetry, with a cytoplasmic half and a nuclear half separated along the NE (Fig. 2). At the center of the IR subunit, two molecules of Nup205 form a homo-dimer, which is flanked by two Nup188 molecules (Fig. 2b, c). Four molecules of Nup93 closely interact with the Nup205/Nup188 hetero-tetramer, with each Nup93 placing an extended helix into the axial groove of Nup205 or Nup188 (Fig. 2c). Together, eight molecules of Nup93, Nup188, and Nup205 assemble into a central scaffold of the IR subunit.

The central scaffold associates with four CNTs on the side of the central pore of the NPC (Fig. 2c). This is accomplished in part by an N-terminal helix of Nup93, which interacts with triple helical bundles of Nup62, Nup58, and Nup54.^13^ The central scaffold also associates with six molecules of Nup155 and two hetero-dimers of ALADIN–NDC1 on the side of the NE. Together, ten molecules of Nup155, ALADIN, and NDC1 assemble into a substructure that has a concave curvature to contact the convex surface of the NE (Fig. 2c). Notably, all six β-propeller domains of Nup155 and two ALADIN β-propellers are located in close proximity to the NE. NDC1 contains a pore domain (PD) and a transmembrane domain (TM). The PD interacts with ALADIN;^14,15^ the TM helps anchor the IR on the NE (Fig. 2c).

For ease of discussion, three Nup155 molecules on the cytoplasmic side are designated Nup155-1, Nup155-2, and Nup155-3 along the cytonuclear axis of the IR subunit (Fig. 2b, c). Similarly, two Nup93 molecules on the cytoplasmic side are referred to as Nup93-1 and Nup93-2, with the latter closer to the center of the IR subunit. Each of the corresponding nucleoporins on the nuclear side is named with an apostrophe. The two halves of the IR subunit are nearly identical to each other, with a root-mean-squared deviation (RMSD) of ~1.34 Å over 8911 Cα atoms (Supplementary information, Fig. S15). Structural comparison of individual nucleoporins between the cytoplasmic and nuclear halves reveals few differences (Supplementary information, Figs. S16, S17). We therefore choose to discuss packing interactions mainly in the cytoplasmic half of the IR subunit.

### Nup188 and Nup205 as the central components

**Fig. 3:**
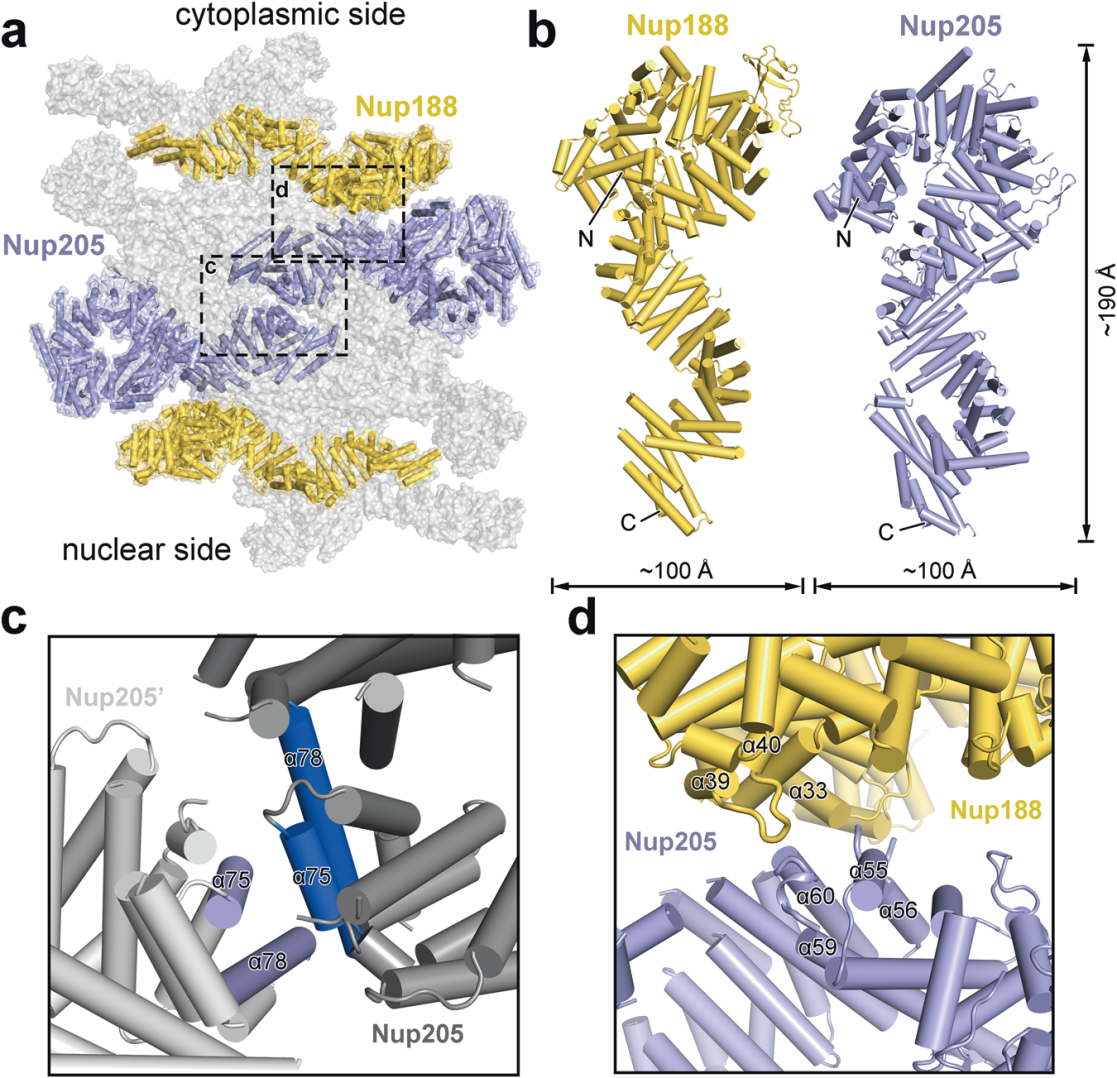
Two molecules of Nup205 and two molecules of Nup188 constitute the central components of the IR subunit. **a** A homo-dimer of Nup205 at the center of the IR subunit is flanked by two molecules of Nup188. The two Nup205 molecules contact each other via their C-terminal helices. **b** A side-by-side comparison of the structures of Nup188 and Nup205. The overall appearance and size are similar between Nup188 (left panel) and Nup205 (right panel). **c** A close-up view on the dimeric interface between Nup205 and Nup205′. The C-terminal helices α75/α78 from Nup205 stack against the corresponding helices from Nup205′. **d** A close-up view on the interface between Nup205 and Nup188. Three inter-helical surface loops from Nup188 and Nup205 are involved in the interactions.

It was unclear whether Nup188 and Nup205 are both present in the IR. Based on the EM map, we conclusively identified two molecules of Nup188 and two molecules of Nup205 in the IR subunit (Fig. 3a; Supplementary information, Fig. S4). Nup188 and Nup205 are similar in both size and overall fold (Fig. 3b). At the center of the IR subunit, Nup205 and Nup205’ form a symmetric homo-dimer that has the appearance of a dumbbell, with their C-terminal helices facing each other at the center (Fig. 3a). Specifically, two helices α75/α78 at the C-terminus of Nup205 stack against two corresponding helices of Nup205′ at a roughly perpendicular angle (Fig. 3c). Nup205 directly associates with Nup188. Specifically, the surface loop between helices α55 and α56 of Nup205 is positioned in close proximity to helix α33 of Nup188 (Fig. 3d). In addition, the surface loop between α39 and α40 of Nup188 is located close to the surface loop between helices α59 and α60 of Nup205.

### Formation of the Nup93–Nup188–Nup205 central scaffold

**Fig. 4:**
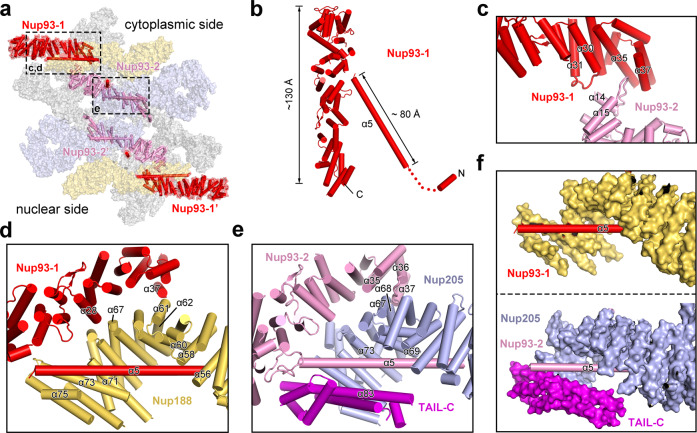
Four molecules of Nup93 interact with Nup188 and Nup205 to form the central scaffold. **a** Overall distribution of four Nup93 molecules in the IR subunit. On the cytoplasmic side, Nup93-1 and Nup93-2 interact with Nup188 and Nup205, respectively. **b** Structure of Nup93. Shown here is a cartoon representation of Nup93-1. The short N-terminal helix is known to interact with CNT.^13^ The extended N-terminal helix α5 binds Nup188 or Nup205. **c** A close-up view on the asymmetric interface between Nup93-1 and Nup93-2. **d** A close-up view on the interface between Nup93-1 and Nup188. Notably, helix α5 of Nup93-1 is placed in the axial groove of the Nup188 α-solenoid and interacts with the C-terminal helices of Nup188. **e** A close-up view on the interface between Nup93-2 and Nup205. In striking analogy to the Nup93-1/Nup188 interface, helix α5 of Nup93-2 is placed in the axial groove of the Nup205 α-solenoid and interacts with the C-terminal helices of Nup205. **f** A side-by-side comparison of the interaction between helix α5 of Nup93 and Nup188 (upper panel) or Nup205 (lower panel). Nup188 and Nup205 are shown in surface representation.

Using the atomic coordinates of Nup93 from the CR subunit,^10^ four molecules were readily placed into the EM map (Fig. 4a; Supplementary information, Fig. S5). Notably, Nup93 contains a short α-helix at the N-terminus and an 80 Å long N-terminal helix α5, which are important for binding CNT and Nup188/Nup205,^12,13^ respectively (Fig. 4a, b). Four Nup93 molecules form two asymmetric pairs, one each on the cytoplasmic and nuclear sides. Although there is no interaction between the two pairs, two Nup93 molecules within the same pair interact with each other. Specifically, the surface loop between helices α30 and α31 of Nup93-1 may contact helix α14 of Nup93-2 (Fig. 4c). In addition, the surface loop between helices α14 and α15 of Nup93-2 is wedged into the crevice formed by helices α35 and α37 from the C-terminal domain (CTD) of Nup93-1.

Our EM map reveals previously unknown structural features in the IR subunit. Remarkably, each of the four Nup93 molecules contains an extended α-helix at its N-terminus (Supplementary information, Fig. S5); this helix α5 traverses the axial groove of the C-terminal portion of the Nup188 or Nup205 α-solenoid (Fig. 4d–f). Specifically, the N-terminal half of helix α5 from Nup93-1 directly contacts helices α56/α58/α60 from Nup188; the C-terminal half of α5 associates with helices α71/α73/α75 from Nup188 (Fig. 4d). In addition, helix α37 of Nup93-1 may contact the surface loop between α61 and α62 of Nup188; helix α28 of Nup93-1 is located close to helix α67 of Nup188.

The interface between Nup93-2 and Nup205 resembles that between Nup93-1 and Nup188. The extended helix α5 from Nup93-2 directly contacts helices α69/α73/α83 from Nup205 (Fig. 4e). In particular, helix α83 comes from the TAIL-C domain,^9^ which nearly forms a closed channel with the Nup205 CTD α-solenoid (Fig. 4f). In contrast, Nup188 lacks the corresponding TAIL-C and the bound helix α5 from Nup93-1 is more surface-exposed (Fig. 4f). In addition, helix α35 of Nup93-2 may contact helix α67 of Nup205 and the surface loop between α36 and α37 of Nup93-2 associates with helices α67/α68 of Nup205 (Fig. 4e).

In summary, these four Nup93 molecules form a network of interfaces with the Nup188–Nup205 hetero-tetramer. Together, these eight nucleoporins constitute a central scaffold of the IR subunit, onto which the CNT and the Nup155–ALADIN–NDC1 are anchored.

### Nup155 links the central scaffold

**Fig. 5:**
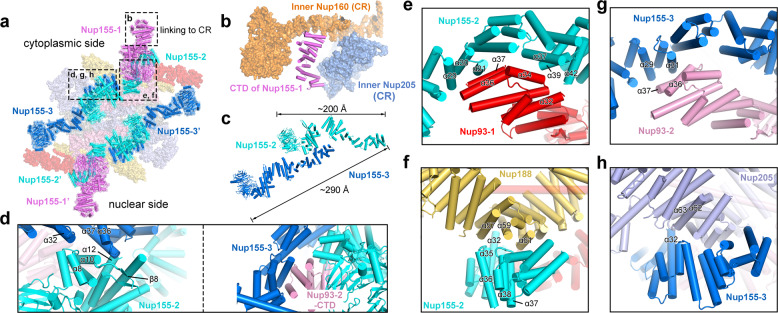
Nup155 links the central scaffold. **a** An overall view of six Nup155 molecules in each IR subunit. Of the six Nup155 molecules, Nup155-2 and Nup155-3 form a dimer on the cytoplasmic side; Nup155-2′ and Nup155-3′ form a homo-dimer on the nuclear side. **b** Nup155 connects the IR subunit to the CR subunit. The C-terminal helices of Nup155 on the cytoplasmic side of the IR subunit are sandwiched by inner Nup160 and inner Nup205 of the CR subunit. **c** A close-up view on the Nup155 homo-dimer. The helical domains of Nup155-2 and Nup155-3 interact with each other in a head-to-tail fashion, generating an elongated Nup155 homo-dimer of ~290 Å in length. **d** Close-up views on the interface between Nup155-2 and Nup155-3. Two views are shown. **e** A close-up view on the interface between Nup155-2 and Nup93-1. **f** A close-up view on the interface between Nup155-2 and Nup188. **g** A close-up view on the interface between Nup155-3 and Nup93-2. This interface is analogous to that between Nup155-2 and Nup93-1. **h** A close-up view on the interface between Nup155-3 and Nup205. This interface is analogous to that between Nup155-2 and Nup188.

Each Nup155 protein contains an N-terminal β-propeller, followed by an elongated α-helical domain. All six Nup155 molecules in the IR subunit are located on the NE side, with their β-propellers directly contacting the NE (Figs. 2c, 5a). Of the six molecules, Nup155-1 and Nup155-1′ directly interact with nucleoporins in the CR and NR, respectively. Specifically, the C-terminal helices of Nup155-1 are sandwiched by inner Nup160 and inner Nup205 from the CR subunit^10^ (Fig. 5b). These interactions may play a major role in the connection between the IR and the CR/NR.

The other four Nup155 molecules form two asymmetric homo-dimers. The elongated α-helical domains of Nup155-2 and Nup155-3 interact with each other in a roughly head-to-tail fashion, creating a homo-dimer of ~290 Å in length (Fig. 5c). Helices α8 and α12 of Nup155-2 directly contact helices α32 and α37 of Nup155-3, respectively; the linker sequence between helix α10 and strand β8 of Nup155-2 is in close proximity to the surface loop between α36 and α37 of Nup155-3 (Fig. 5d, left panel). Notably, the CTD of Nup93-2 associates with both Nup155-2 and Nup155-3, likely stabilizing their interface^16^ (Fig. 5d, right panel).

This asymmetric dimer interacts with all three components of the central scaffold. The C-terminal helices of Nup155-2 closely stack against the C-terminal helices of Nup93-1 and the middle portion of Nup188. On one hand, helices α29/α30/α31, α37/α39, and α42 of Nup155-2 may contact helices α36/α37, α34, and α32 of Nup93-1, respectively (Fig. 5e). On the other hand, the loop between helices α35/α36, helix α32, and the loop between α37/α38 of Nup155-2 interact with helices α57, α59, and α61 of Nup188, respectively (Fig. 5f).

The interactions involving Nup155-3 closely resemble those involving Nup155-2. Nup155-3 stacks against Nup93-2 and Nup205. On one hand, helices α29/α31 of Nup155-3 interact with helices α36/α37 of Nup93-2 (Fig. 5g). On the other hand, helix α32 of Nup155-3 may directly contact the surface loop between helices α62 and α63 (Fig. 5h).

### The ALADIN–NDC1 dimer

**Fig. 6:**
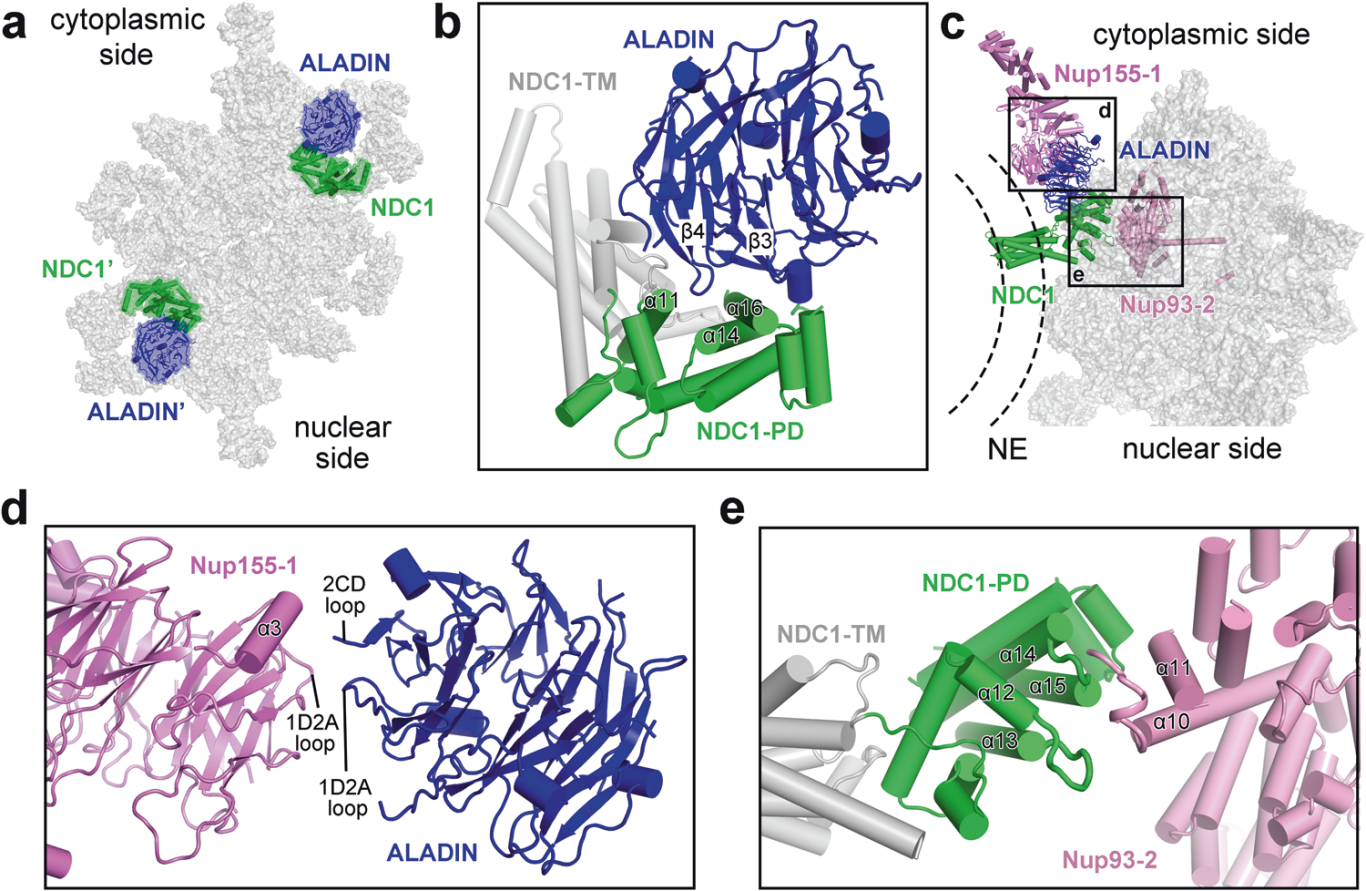
The ALADIN–NDC1 hetero-dimer helps to anchor the IR subunit to the NE. **a** An overall view of two ALADIN–NDC1 hetero-dimers in the IR subunit. **b** A close-up view on the ALADIN–NDC1 hetero-dimer. Blade-3 and blade-4 of the ALADIN β-propeller are in close proximity to helices α11/α14/α16 of the PD of NDC1. **c** An overall view on the cytoplasmic ALADIN–NDC1 hetero-dimer relative to its neighboring nucleoporins Nup155 and Nup93. **d** A close-up view on the interface between ALADIN and Nup155-1. **e** A close-up view on the interface between NDC1 and Nup93-2.

The nucleoporin ALADIN is thought to directly interact with NDC1 in human cells.^14,15^ However, the exact locations of ALADIN and NDC1 in the IR subunit have remained enigmatic. In two regions of the EM map next to Nup155-1 and Nup155-1′, there are two lobes of significant density (Fig. 6a; Supplementary information, Fig. S7). Unfortunately, these regions are in the periphery of the IR subunit where the quality of the EM density is insufficient for ab initio modeling of nucleoporins. Using AlphaFold,^11^ we generated a predicted structure of the *X. laevis* NDC1; the PD of NDC1 fits the EM map quite well (Supplementary information, Fig. S7). Next, we generated an AlphaFold-predicted structure of the ALADIN–NDC1 hetero-dimer, which, to our pleasant surprise, can be docked into the EM density map with little adjustment (Supplementary information, Fig. S7).

There are two ALADIN–NDC1 hetero-dimers in each IR subunit, one on the cytoplasmic side and the other on the nuclear side (Fig. 6a). In the modeled structure, the loop between strands C and D of blade 4 (known as the 4CD loop) from the ALADIN β-propeller is in close proximity to helices α11 and α14 of NDC1-PD (Fig. 6b). In addition, the strands C and D of blade 3 (β3C and β3D) and the intervening 3AB loop from ALADIN may contact helix α16 of NDC1-PD.

The ALADIN–NDC1 hetero-dimer bridges the gap between Nup155-1 and Nup93-2 in the cytoplasmic half of the IR subunit (Fig. 6c). Specifically, the 1D2A loop and the 2CD loop of the ALADIN β-propeller are positioned close to the 1D2A loop and helix α3 of the N-terminal β-propeller of Nup155-1, respectively (Fig. 6d). Two surface loops from NDC1-PD, one between helices α12 and α13 and the other between α14 and α15, are located close to the surface loop between helices α10 and α11 of Nup93-2 (Fig. 6e).

The ALADIN–NDC1 hetero-dimer appears to play an important role in connecting Nup155-1 to the IR subunit. Nup155-1 and Nup155-1′, on the other hand, connect the IR to the CR and the NR, respectively (Fig. 5a, b). Any mutation in ALADIN that compromises its structural stability or its interaction with NDC1 is likely to have a negative impact on the connection of the IR with the CR/NR, which may consequently damage assembly and function of the NPC.

### Formation of the IR

**Fig. 7:**
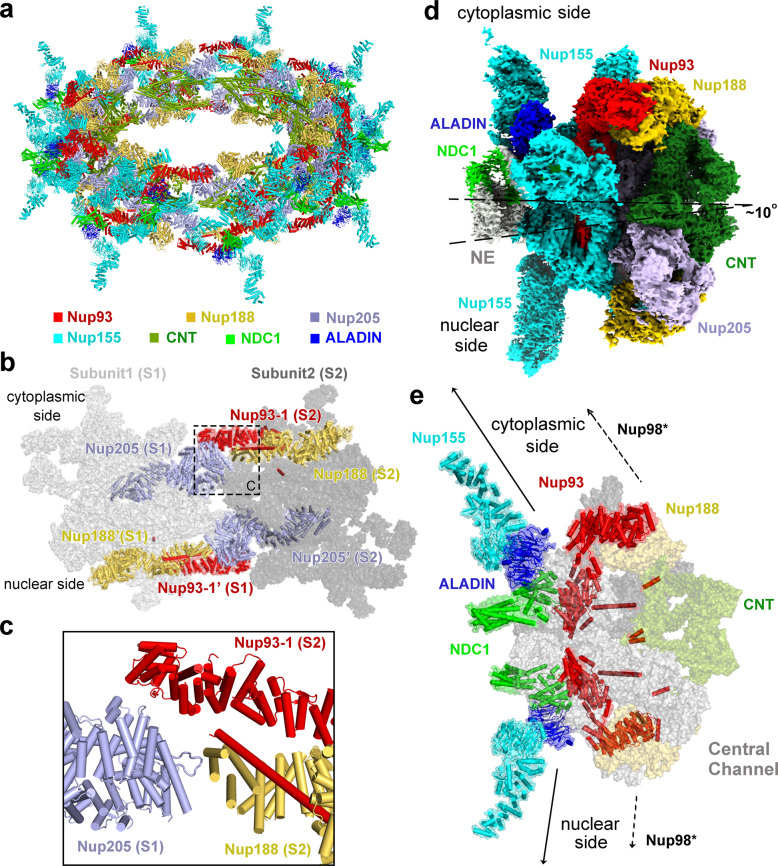
Structural features of the IR scaffold. **a** An overall view of the IR scaffold. **b** An overall view on inter-subunit interface between subunit 1 (S1) and subunit 2 (S2). **c** A close-up view on the cytoplasmic side of the interface between S1 and S2. Given the spatial gap in between, Nup205 of S1 may not directly interact with Nup93-1 or Nup188 from S2. **d** The IR subunit is slightly tilted toward the cytoplasmic side. **e** The IR subunit is linked to the outer rings through the linker nucleoporins Nup93, NDC1, ALADIN, and Nup155-1. The IR subunit is connected in opposite directions to the outer rings. Such connection depends on the ALADIN–NDC1 hetero-dimer to fill up the vacancy between Nup155-1 and Nup93-2.

We first aligned the 4.2 Å reconstruction of the IR subunit onto the EM map of the IR at 22 Å resolution. This practice was repeated eight times to generate a complete reconstruction of the IR. We then projected the atomic coordinates of the IR subunit into the reconstruction for each of the eight IR subunits, generating a composite model for the entire IR scaffold (Fig. 7a). This composite model contains 240 nucleoporins: 48 copies of Nup155, 32 copies each of Nup93 and CNT, and 16 copies each of Nup188, Nup205, ALADIN, and NDC1. The IR components Nup98 and Nup35 remain to be identified.

The inter-subunit connection in the IR scaffold mainly involves Nup205 from subunit 1 (S1) and Nup93-1 and Nup188 from subunit 2 (S2) (Fig. 7b). The N-terminal helices of Nup205 from S1 are located close to the N-terminal helices of Nup93-1 and the C-terminal helices of Nup188 from S2 (Fig. 7c). Notably, however, the spatial gap between these potentially interacting helices from S1 and S2 is large enough to disallow direct interactions. This analysis suggests a weak interface between neighboring IR subunits, which is consistent with the observed contraction and dilation of the NPC pore.^17–19^ The small and large spatial gaps between two neighboring IR subunits may correspond to the contracted and dilated states of the NPC pore.^19–21^

Unlike CR or NR, the IR has a nearly perfect internal symmetry (Supplementary information, Fig. S15). However, the symmetric IR subunit is placed on the NE asymmetrically, tilting toward the cytoplasmic side by ~10° (Fig. 7d). The IR subunit is connected to the CR/NR through the linker nucleoporins Nup155, ALADIN, NDC1, and Nup93 (Fig. 7d). Such connection depends on the ALADIN–NDC1 hetero-dimer, which fills up the spatial gap between Nup155 and Nup93.

## Discussion

The structural model of vertebrate IR subunit reported here may serve as a framework for understanding the functional mechanism of NPC. A number of the nucleoporins are regulated by phosphorylation, which impacts on the assembly and function of NPC.^22,23^ We mapped the phosphorylated sites onto the structure^24,25^ (Supplementary information, Fig. S18). A number of phosphorylation sites are located to inter-nucleoporin interfaces or in close proximity to the NPC–NE interface; phosphorylation of these residues may play key roles in disrupting the architecture of nuclear pore upon onset of NPC disassembly. Ser307 and Ser675 of Nup155 face the nuclear membrane; phosphorylation of either residue is likely to alter their interaction with the membrane, thus potentially affecting membrane localization of Nup155 (Supplementary information, Fig. S18a). Phosphorylation of Ser117 in helix α5 of Nup93 may affect its interaction with Nup205 or Nup188; phosphorylation of Thr368 or Ser655 of Nup93 may alter the interface between Nup93-1 and Nup93-2 (Supplementary information, Fig. S18b). Analogously, phosphorylation of Nup188, Nup205 and CNT may change their local conformation or local environment for interaction (Supplementary information, Fig. S18c). These structural observations are also in line with those observed in the yeast NPC.^21,26^

ALADIN is regulated by phosphorylation and is frequently targeted for mutation in the triple A syndrome (AAAS).^27–29^ All three potential phosphorylation sites (Ser186, Thr295, and Ser300) in ALADIN^24,25^ are located on surface loops on the top side of the β-propeller (Supplementary information, Fig. S19a); these residues are likely involved in protein recruitment or interaction that might be sabotaged by mutations. Consistent with this analysis, this side of the ALADIN β-propeller is targeted by 15 AAAS-derived mutations^30–43^ (Supplementary information, Fig. S19b). Presumably, these mutations either alter the structure or damage the interaction of ALADIN with other proteins. Other nucleoporins have been found to be targeted for mutations in different diseases, including Nup93 and Nup205 in steroid-resistant nephrotic syndrome (SRNS)^44^ and Nup62 in infantile bilateral striatal necrosis (IBSN)^45^ (Supplementary information, Fig. S20a, b). For example, replacement of Gln416 by a helix-breaking residue Pro in the middle of an extended helix of Nup62 may destabilize its helical conformation (Supplementary information, Fig. S20b).

As a component of the central scaffold, Nup93 plays a key role in organizing the IR subunit through direct interactions with multiple nucleoporins^16,46^ (Fig. 4d–f). Remarkably, the interaction between Nup93-α5 and Nup205 axial groove in the IR subunit is exactly preserved in the CR subunit.^10^ A close examination of the Nup93–Nup205 interactions reveals an RMSD of no more than 4 Å over more than 1200 aligned Cα atoms between the IR and CR subunits (Supplementary information, Fig. S21).

The fact that the same helix α5 of Nup93 interacts similarly with Nup205 or Nup188 in both the CR and IR subunits suggests remarkable adaptability of Nup93. Indeed, the inherent conformational flexibility of Nup93 allows it to bridge interactions with a number of protein components in both the outer rings and the IR. Such conformational flexibility is a hallmark of several other nucleoporins, as exemplified by Nup155 (Supplementary information, Fig. S22). Although the two corresponding molecules of Nup155 in the cytoplasmic and nuclear halves are structurally identical (Supplementary information, Fig. S22a), different Nup155 molecules on the same side exhibit very large RMSD values (Supplementary information, Fig. S22b, c). Interestingly, the role of Nup155-2 and Nup155-3 in the IR subunit is filled by two different molecules (Nup157 and Nup170) in the yeast NPC.^21,26^ This further highlights the importance of Nup155 conformational flexibility in IR assembly. Moreover, protozoa such as yeast undergo closed mitosis, as opposed to open mitosis in which the NE collapses. Whether the exceptionally high conformational flexibility of Nup155 is linked to the necessity of NPC disassembly during mitosis remains to be investigated.

Despite improvement in the quality of EM reconstruction for the vertebrate IR subunit, the average resolution of our EM reconstruction falls just short of the atomic range. The local resolution drops off quickly in the peripheral regions of the IR subunit. Consequently, we have been unable to identify the RRM-containing nucleoporin Nup35 or the linker nucleoporin Nup98. The current EM reconstruction suggests potential locations of Nup35 (Supplementary information, Fig. S23). Further improved resolution should allow definitive assignment of Nup35 and Nup98.

In summary, we have determined the cryo-EM reconstruction of the *X. laevis* IR subunit at an average resolution of 4.2 Å, the highest achieved for the IR subunit of a vertebrate NPC. Structural analysis reveals a wealth of structural features that will facilitate an advanced mechanistic understanding of NPC assembly and function.

## Materials and methods

### Cryo-EM sample preparation and EM data acquisition

The same cryo-EM datasets used for reconstruction of the CR subunit from *X. laevis* oocytes^10^ were used for reconstruction of the IR subunit. In short, the NE from *X. laevis* oocytes was prepared as described.^5,9^ The cryo-EM sample was prepared as described.^10^ The gold EM grids (R1.2/1.3&R2/1&R2/2; Quantifoil, Jena, Germany) were blotted for 8 s with a blot force of 15 and vitrified by plunge-freezing into liquid ethane using a Vitrobot Mark IV (Thermo Fisher Scientific) at 8 °C under 100% humidity.

Details of data acquisition are as described.^10^ With the grids tilting at angles of 0°, 30°, 45°, and 55°, 46,143 micrographs were recorded on a Titan Krios electron microscope (FEI) operating at 300 kV with a nominal magnification of 64,000× and equipped with a Gatan GIF Quantum energy filter (slit width 20 eV) (Supplementary information, Table S1). A K3 detector (Gatan Company) was used in the super-resolution mode, with a calibrated pixel size of 0.6935 Å for the movie files (Supplementary information, Table S1). The movie images were binned twice during motion correction, arriving at a pixel size of 1.387 Å for the final motion corrected images. All frames in each stack were first aligned and summed using MotionCor2.^47^ Dose weighting was performed using MotionCor2.^47^ The average defocus values were set between –1.5 and –3.0 μm and were estimated using Gctf.^48^

### An initial model of the IR from *X. laevis* oocytes

33,747 micrographs were manually selected from the original dataset of 46,143 micrographs. A total of 800,825 particles were manually selected from these micrographs (Supplementary information, Fig. S1a). Initial defocus estimation was carried out as previously described^9^ prior to all other data processing procedures.

The central portion of an NPC comprises four ring scaffolds: CR, IR, NR, and LR. Due to the inherent flexibility among the four rings, it is practically impossible to refine the entire NPC as a single particle to high resolution. We therefore carried out initial pose estimation of the NPC particles on one of the relatively stable ring scaffolds: the CR. The NPC particles were first aligned to the CR side as described.^10^ Following refinement of the CR structure, we continued the 3D refinement procedure from the last iteration with a layered mask focusing on the IR and LR layer. Only pixels within a specific layer, with *z*_start < *z* < *z*_end, have pixel values of 1; all other pixels that have *z* coordinates below or above this layer have zero values. Transition between the two regions follows a raised cosine scheme. The continued refinement yielded a reconstruction at 22 Å resolution based on 660,302 NPC particles (Supplementary information, Fig. S1a).

The resulting data star files from the final round of auto-refinement were imported into cryoSparc^49^ for one round of Local-Refinement to obtain a reconstruction at 22 Å resolution (Supplementary information, Fig. S1a). The nominal resolution is the same as that reported in RELION^50^ but the EM map has substantially reduced noises in the CR region (Supplementary information, Fig. S1a). The C8 symmetry was applied throughout this stage of data processing. The IR from *X. laevis* exhibits an inner diameter of 45 nm, close to that reported in human NPC from purified NE^51^ (Supplementary information, Fig. S1b). However, this inner diameter is significantly constricted when compared to that reported in the in situ structures of human NPC.^19,52^

### Data processing and reconstruction of the IR subunit

We extracted the IR subunit particles based on the alignment parameters of the 22 Å IR reconstruction. We updated the orientation, shift and defocus parameters for each subunit according to a published protocol.^9^ 5,223,773 particles of the IR subunit were extracted using a box size of 128 and a binned pixel size of 5.548 Å (Supplementary information, Fig. S2a). We performed one round of 3D classification (K = 1) with ten iterations. The data star file from iteration 10 was then used for re-extraction of bin2 particles with a box size of 256 and a binned pixel size of 2.774 Å (Supplementary information, Fig. S2a). The entire dataset of bin2 particle of the IR subunit was then subjected to three rounds of parameter refinement.^10^ This practice allowed selection of 2,139,754 particles, which yielded a reconstruction of the IR subunit at 5.6 Å resolution.

To fully utilize the dataset, these IR subunits were projected back to the original IR particles. The defocus values of all subunit particles within the same IR were pooled together to calculate a corrected average defocus value for the center of mass of each IR particle. All subunits of this IR were then re-extracted using the updated defocus value deduced from the corrected average defocus of this IR particle. Through this procedure, 3,013,260 subunit particles were extracted, resulting in a reconstruction at 5.6 Å resolution after auto-refinement (Supplementary information, Fig. S2a).

This dataset was then used for re-extraction with a box size of 400 and pixel size of 1.387 Å (Supplementary information, Fig. S2b). Data processing beyond this point was carried out as described.^9^ In short, the extracted particles were directly subjected to one round of CTF-refinement followed by one round of auto-refinement, resulting in a reconstruction of the IR subunit at 5.2 Å resolution (Supplementary information, Fig. S2b). Three additional rounds of parameter refinement cycles were repeated to arrive at a final average resolution of 4.2 Å from 2,093,631 particles, using a mask that masks out the NE and regions from adjacent subunits (Supplementary information, Fig. S2b). The angular distribution of the particles is reasonable and the overall reconstruction has isotropic resolution (Supplementary information, Fig. S3). The final EM map displays clear features for secondary structural elements (Supplementary information, Figs. S4–S8). These features facilitated sequence assignment of the nucleoporins (Supplementary information, Figs. S9–S14).

### Atomic modeling of the IR subunit

The previous EM map of the IR subunit from human NPC, achieved through cryo-ET analysis, displays an average resolution of ~21 Å.^8^ The atomic coordinates of human IR (PDB: 5IJO) were manually fitted into our 4.2 Å reconstruction of the *X. laevis* IR subunit using Chimera.^53^ The much improved EM density maps allowed unambiguous assignment of most IR components and accurate placement of secondary structural elements. This practice allows identification of 30 molecules of nucleoporins in each IR subunit, including four copies of Nup93, six copies of Nup155, four CNTs (composed of Nup62, Nup58 and Nup54),^13^ two copies of NDC1, two copies of ALADIN, two copies of Nup205 and two copies of Nup188.

The atomic coordinates of Nup93 and Nup205 from the CR subunit^10^ were docked into the EM maps with little adjustment. For the placement of the N-terminal helix α2 of Nup93, the crystal structure of *Chaetomium thermophilum* CNT bound to Nic96 (PDB: 5CWS) was used for modeling Nup93-α2 into the lowpass filtered map. Using the recently released structure prediction tool AlphaFold,^11^ we generated atomic coordinates for each of the other *X. laevis* proteins. The predicted structures for most *X. laevis* nucleoporins are remarkably similar to the homology-modeled structures of the corresponding human nucleoporins. The predicted structure of the CNT (Nup62, Nup58 and Nup54) is nearly identical to that of the reported structure for *X. laevis* CNT (PDB: 5C3L). We individually docked each predicted structure of the *X. laevis* nucleoporin into the 4.2 Å EM map by Chimera^53^ and made manual adjustment according to features of secondary structural elements using Coot.^54^ The final atomic model of the *X. laevis* IR subunit contains 18,742 amino acids, with 772 α-helices and 296 β-strands.

## Supplementary information

Supplementary information, Fig. S1

Supplementary information, Fig. S2

Supplementary information, Fig. S3

Supplementary information, Fig. S4

Supplementary information, Fig. S5

Supplementary information, Fig. S6

Supplementary information, Fig. S7

Supplementary information, Fig. S8

Supplementary information, Fig. S9

Supplementary information, Fig. S10

Supplementary information, Fig. S11

Supplementary information, Fig. S12

Supplementary information, Fig. S13

Supplementary information, Fig. S14

Supplementary information, Fig. S15

Supplementary information, Fig. S16

Supplementary information, Fig. S17

Supplementary information, Fig. S18

Supplementary information, Fig. S19

Supplementary information, Fig. S20

Supplementary information, Fig. S21

Supplementary information, Fig. S22

Supplementary information, Fig. S23

Supplementary information, Table S1

Supplementary information, Table S2

Supplementary information, Table S3

## Data Availability

The atomic coordinates of the IR subunit have been deposited in the Protein Data Bank with the accession code 7WKK. The EM map for the IR subunit has been deposited in the EMDB with the accession code EMD-32566.
