# Supplementary material for: Cryo-EM structure of the inner ring from the Xenopus laevis nuclear pore complex
Source: Cell Res. 2022 Mar 18;32(5):451–60. doi: 10.1038/s41422-022-00633-x (PMC9061766; doi:10.1038/s41422-022-00633-x)
Supplement: Supplementary file 1 — Supplementary information, Fig. S1 [file 41422_2022_633_MOESM1_ESM.pdf]

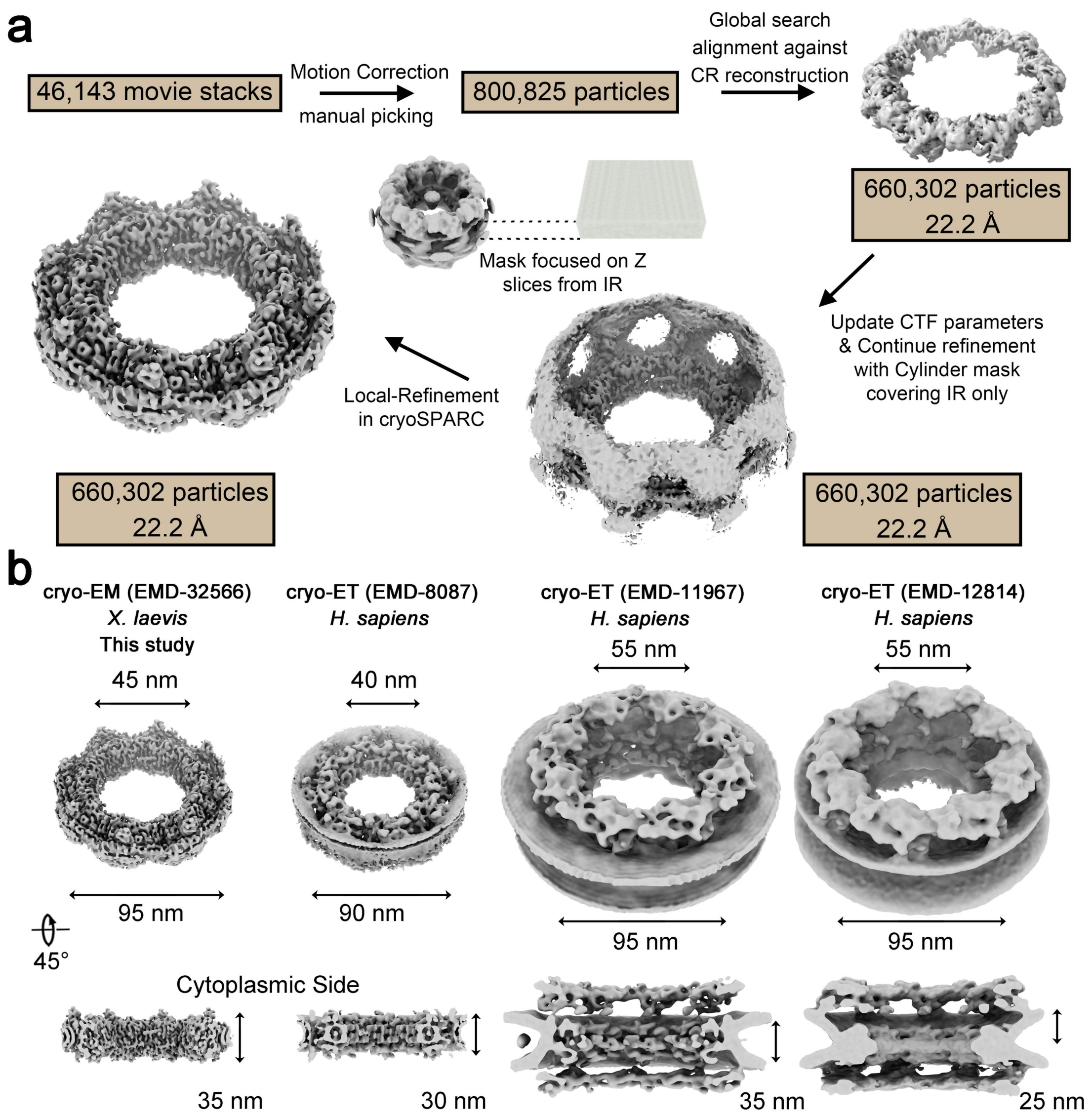

Supplementary information, Fig. S1 | Cryo-EM analysis of the IR from the *X. laevis* NPC.

a, Flowchart of data analysis for reconstruction of the IR from the *X. laevis* NPC at 22 Å resolution. **b**, Structural comparison of the IR from the *X. laevis* NPC determined using single particle cryo-EM with that of the human NPC obtained through cryo-ET. The EMDB codes for the 3D maps, including the one for this study, are shown on top.
