# Supplementary material for: Cryo-EM structure of the inner ring from the Xenopus laevis nuclear pore complex
Source: Cell Res. 2022 Mar 18;32(5):451–60. doi: 10.1038/s41422-022-00633-x (PMC9061766; doi:10.1038/s41422-022-00633-x)
Supplement: Supplementary file 2 — Supplementary information, Fig. S2 [file 41422_2022_633_MOESM2_ESM.pdf]

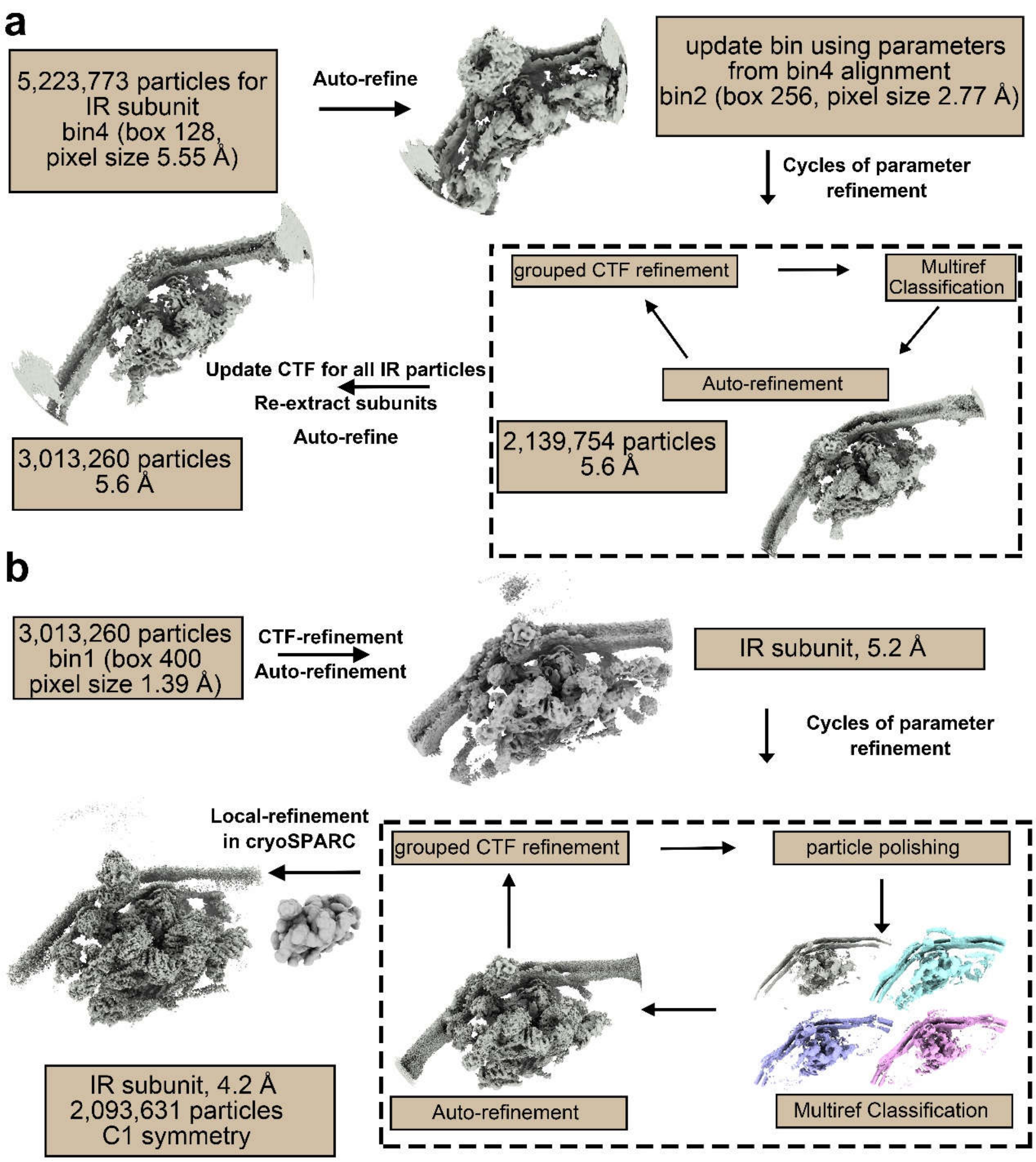

Supplementary information, Fig. S2 | Cryo-EM data processing for the IR subunit from *X. laevis* NPC.

a, Flowchart of data processing for reconstruction of the IR subunit to an overall resolution of 5.6 Å. This part of the data analysis only involves the bin-4 (pixel size 5.548 Å) and bin-2 levels (pixel size 2.774 Å). **b**, Flowchart of data processing for the IR subunit to an overall resolution of 4.2 Å. This part of the data analysis involves the bin-1 level (pixel size 1.387 Å). Please refer to the section "Data processing and reconstruction of the IR subunit" in the MATERIALS AND METHODS for details.
