# Supplementary material for: Cryo-EM structure of the inner ring from the Xenopus laevis nuclear pore complex
Source: Cell Res. 2022 Mar 18;32(5):451–60. doi: 10.1038/s41422-022-00633-x (PMC9061766; doi:10.1038/s41422-022-00633-x)
Supplement: Supplementary file 3 — Supplementary information, Fig. S3 [file 41422_2022_633_MOESM3_ESM.pdf]

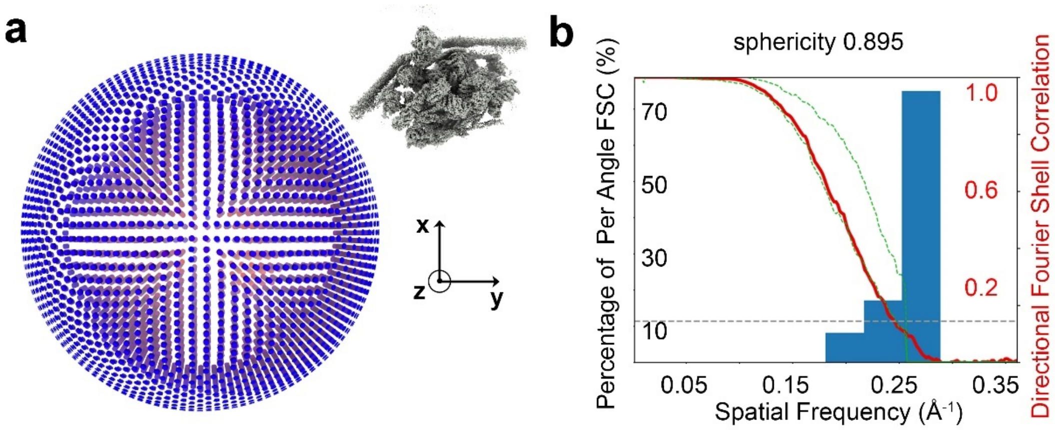

Supplementary information, Fig. S3 | Cryo-EM analysis of the IR subunit.
a, Angular distribution of the single-particle cryo-EM reconstruction for the IR subunit. Each cylinder represents one view and the height of the cylinder is proportional to the number of particles for that view. **b**, Directional Fourier Shell correlation (FSC) curves and directional FSC histograms for cryo-EM reconstruction of the IR subunit. The directional FSC curve was calculated using the website https://3dfsc.salk.edu[1].

Reference

1 Tan YZ, Baldwin PR, Davis JH et al. Addressing preferred specimen orientation in single-particle cryo-EM through tilting. Nat Methods 2017; 14:793-796.
