# Supplementary material for: Cryo-EM structure of the inner ring from the Xenopus laevis nuclear pore complex
Source: Cell Res. 2022 Mar 18;32(5):451–60. doi: 10.1038/s41422-022-00633-x (PMC9061766; doi:10.1038/s41422-022-00633-x)
Supplement: Supplementary file 4 — Supplementary information, Fig. S4 [file 41422_2022_633_MOESM4_ESM.pdf]

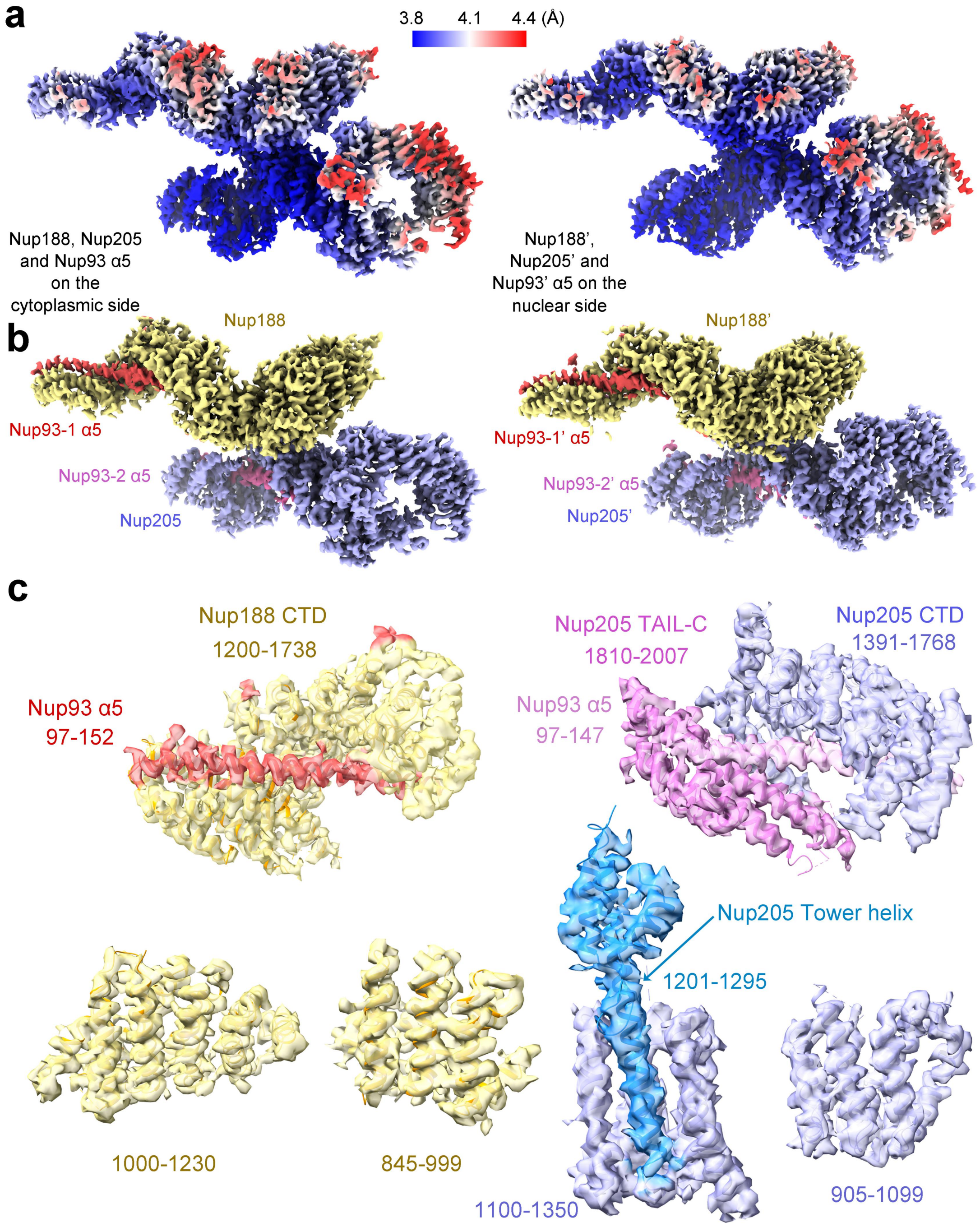

Supplementary information, Fig. S4 | EM maps and model fitting for Nup188 and Nup205.

a, Resolution maps for the two copies of Nup188 and Nup205. Nup188 and Nup205 on the cytoplasmic side (left) and on the nuclear side (right) are nearly identical. The local resolution maps are calculated in Relion 3.0 and presented in Chimera. **b**, EM maps for the two copies of Nup188, Nup205, and the extended helices α5 from Nup93 that respectively bind to them. The same views are shown as in panel a. **c**, Model building of Nup188 and Nup205 into their EM corresponding densities. The EM maps, shown as semitransparent surface in ChimeraX, are color coded for different proteins or domains. Nup205 specific TAIL-C and tower helix are highlighted in magenta (top

right) and blue (bottom right), respectively. All EM maps in this figure were prepared using the 4.2-Å reconstruction of the IR subunit with a contour level of 5-7 σ.
