# Supplementary material for: Cryo-EM structure of the inner ring from the Xenopus laevis nuclear pore complex
Source: Cell Res. 2022 Mar 18;32(5):451–60. doi: 10.1038/s41422-022-00633-x (PMC9061766; doi:10.1038/s41422-022-00633-x)
Supplement: Supplementary file 5 — Supplementary information, Fig. S5 [file 41422_2022_633_MOESM5_ESM.pdf]

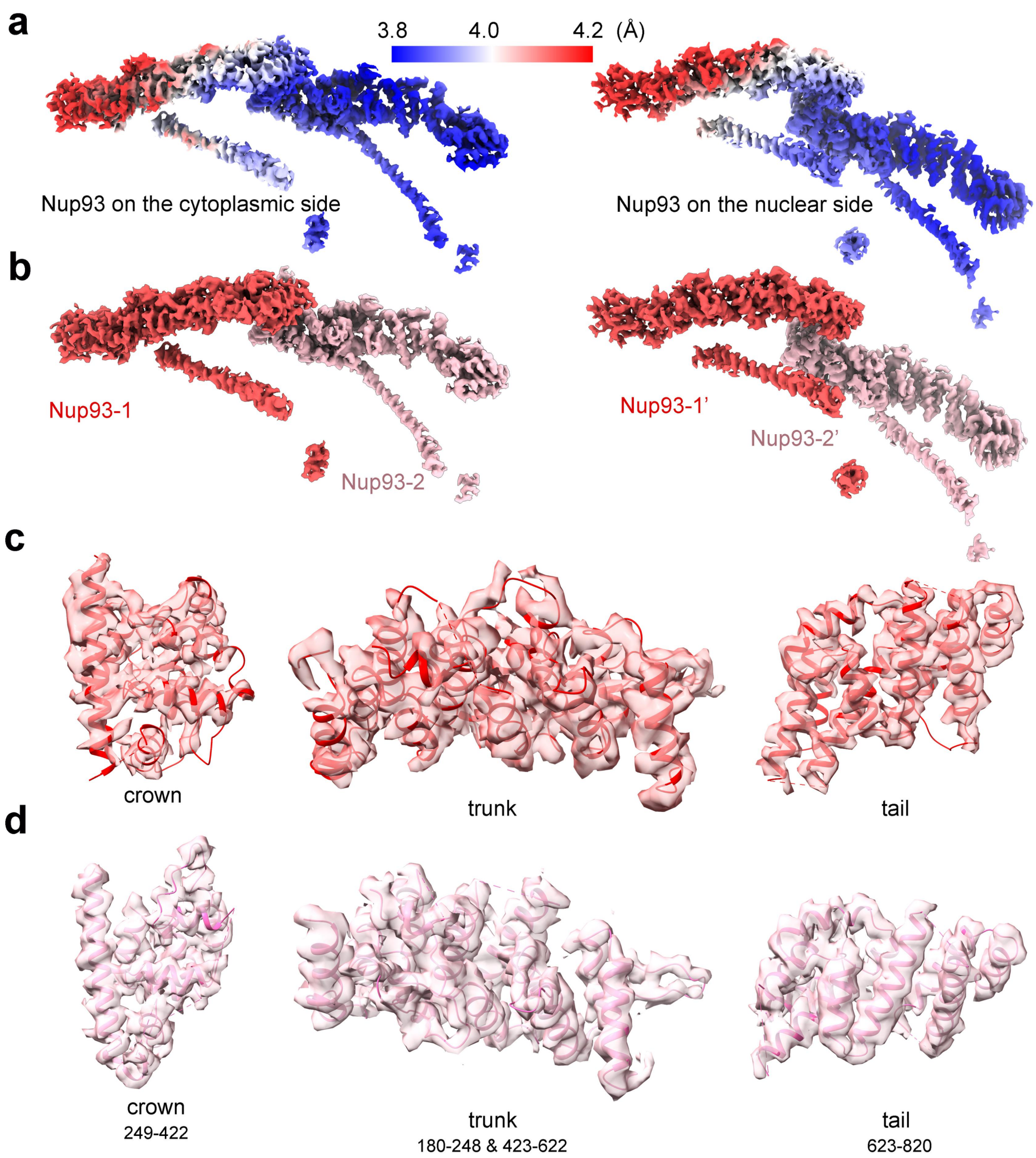

Supplementary information, Fig. S5 | EM maps and model fitting for Nup93.

a, Resolution maps for the two copies of Nup93 pairs. Nup93 pairs on the cytoplasmic side (left) and on the nuclear side (right) are nearly identical. The local resolution maps are calculated in Relion 3.0 and presented in Chimera. **b**, EM maps for the two copies of Nup93 pairs. The views are identical to the corresponding ones in panel a. **c**,**d,** Model building of Nup93-1 and -2 into their corresponding densities. The EM maps, shown as semitransparent surface, were prepared using the 4.2-Å reconstruction of the IR subunit with a contour level of 5-7 σ in ChimeraX.
