# Supplementary material for: Cryo-EM structure of the inner ring from the Xenopus laevis nuclear pore complex
Source: Cell Res. 2022 Mar 18;32(5):451–60. doi: 10.1038/s41422-022-00633-x (PMC9061766; doi:10.1038/s41422-022-00633-x)
Supplement: Supplementary file 6 — Supplementary information, Fig. S6 [file 41422_2022_633_MOESM6_ESM.pdf]

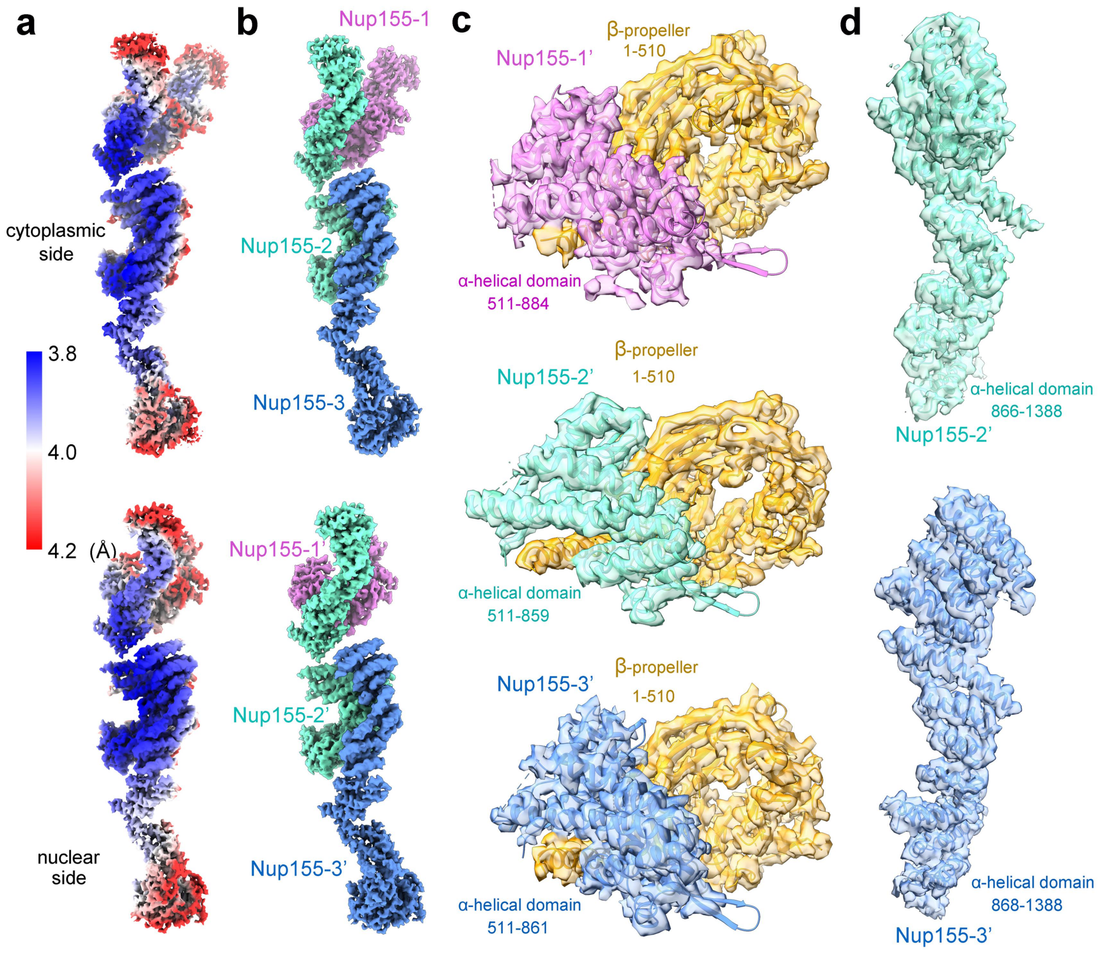

Supplementary information, Fig. S6 | EM maps and model fitting for Nup155. **a**, Resolution maps for the two copies of the Nup155 trimer. Nup155 trimers on the cytoplasmic side (top) and nuclear side (bottom) are nearly identical. The local resolution maps are calculated in Relion 3.0 and presented in Chimera. **b**, Color-coded EM maps for the two copies of the Nup155 trimer. **c**, Model building of the NTD of Nup155 into their EM densities. **d**, Model building of the CTD of Nup155 into their EM densities. The EM maps, shown as semitransparent surface in ChimeraX, are color coded based on the protein identity. All EM maps in this figure were prepared using the 4.2-Å reconstruction of the IR subunit with a contour level of 4-6 σ.
