# Supplementary material for: Cryo-EM structure of the inner ring from the Xenopus laevis nuclear pore complex
Source: Cell Res. 2022 Mar 18;32(5):451–60. doi: 10.1038/s41422-022-00633-x (PMC9061766; doi:10.1038/s41422-022-00633-x)
Supplement: Supplementary file 7 — Supplementary information, Fig. S7 [file 41422_2022_633_MOESM7_ESM.pdf]

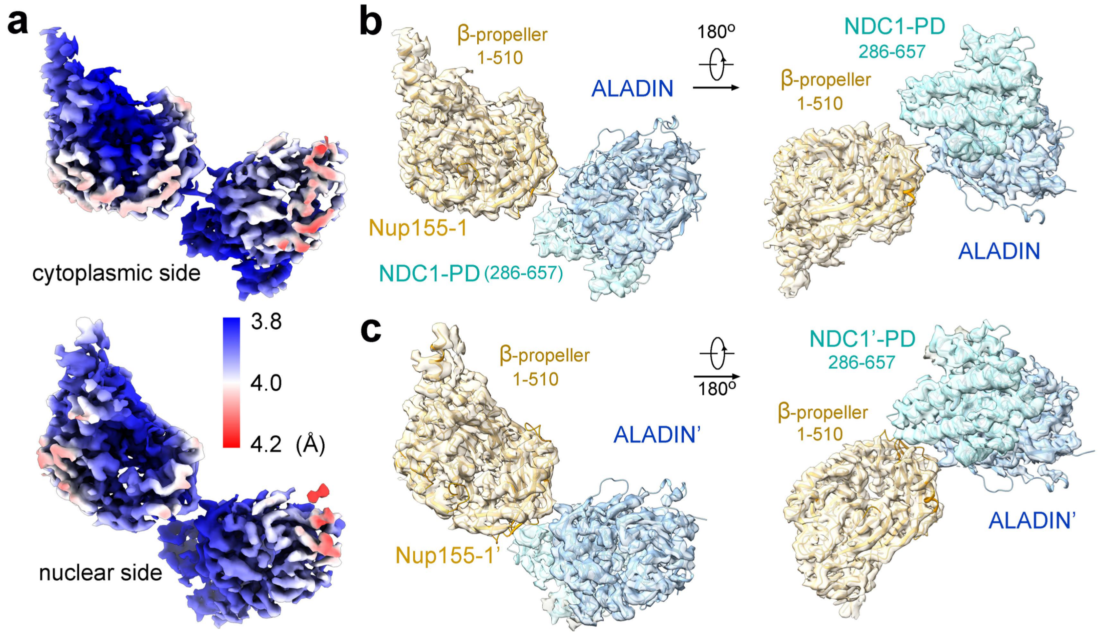

Supplementary information, Fig. S7 | EM maps and model fitting for β-propeller of Nup155, ALADIN, and NDC1.

a, Resolution maps for the two copies of the Nup155-ALADIN-NDC1 trimer. The Nup155-ALADIN-NDC1 trimers on the cytoplasmic side (top) and on the nuclear side (bottom) are nearly identical. The local resolution maps are calculated in Relion 3.0 and presented in Chimera. **b**,**c,** Model building of Nup155-ALADIN-NDC1 trimers into their corresponding EM maps. The color-coded EM maps, shown as semitransparent surface in ChimeraX, were prepared using the 4.2-Å reconstruction of the IR subunit with a contour level of 4-6 σ.
