# Supplementary material for: Cryo-EM structure of the inner ring from the Xenopus laevis nuclear pore complex
Source: Cell Res. 2022 Mar 18;32(5):451–60. doi: 10.1038/s41422-022-00633-x (PMC9061766; doi:10.1038/s41422-022-00633-x)
Supplement: Supplementary file 8 — Supplementary information, Fig. S8 [file 41422_2022_633_MOESM8_ESM.pdf]

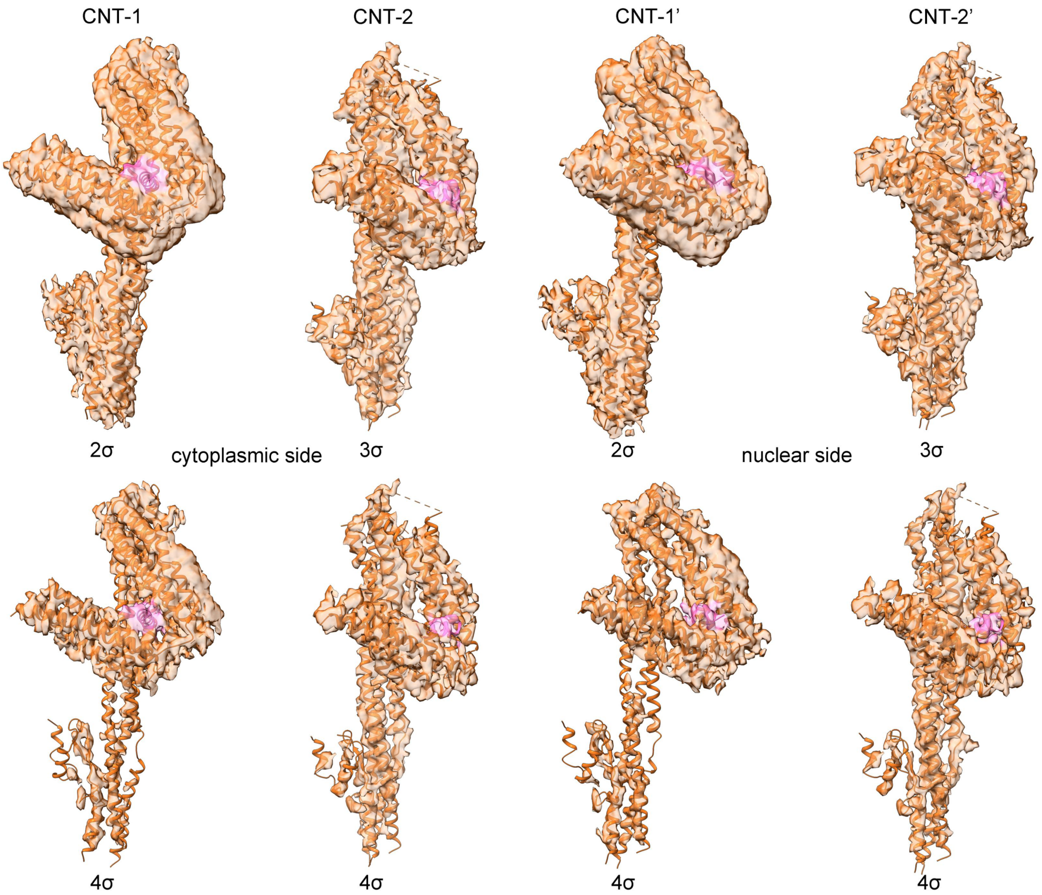

Supplementary information, Fig. S8 | Model fitting for CNTs.
Semitransparent contour of the EM maps docked with structural models of CNTs are shown in Chimera. The same views are shown with low and high thresholds on the top and bottom, respectively. CNT and the N-terminal helix from Nup93 are colored orange and pink, respectively.
