# Supplementary material for: Cryo-EM structure of the inner ring from the Xenopus laevis nuclear pore complex
Source: Cell Res. 2022 Mar 18;32(5):451–60. doi: 10.1038/s41422-022-00633-x (PMC9061766; doi:10.1038/s41422-022-00633-x)
Supplement: Supplementary file 9 — Supplementary information, Fig. S9 [file 41422_2022_633_MOESM9_ESM.pdf]

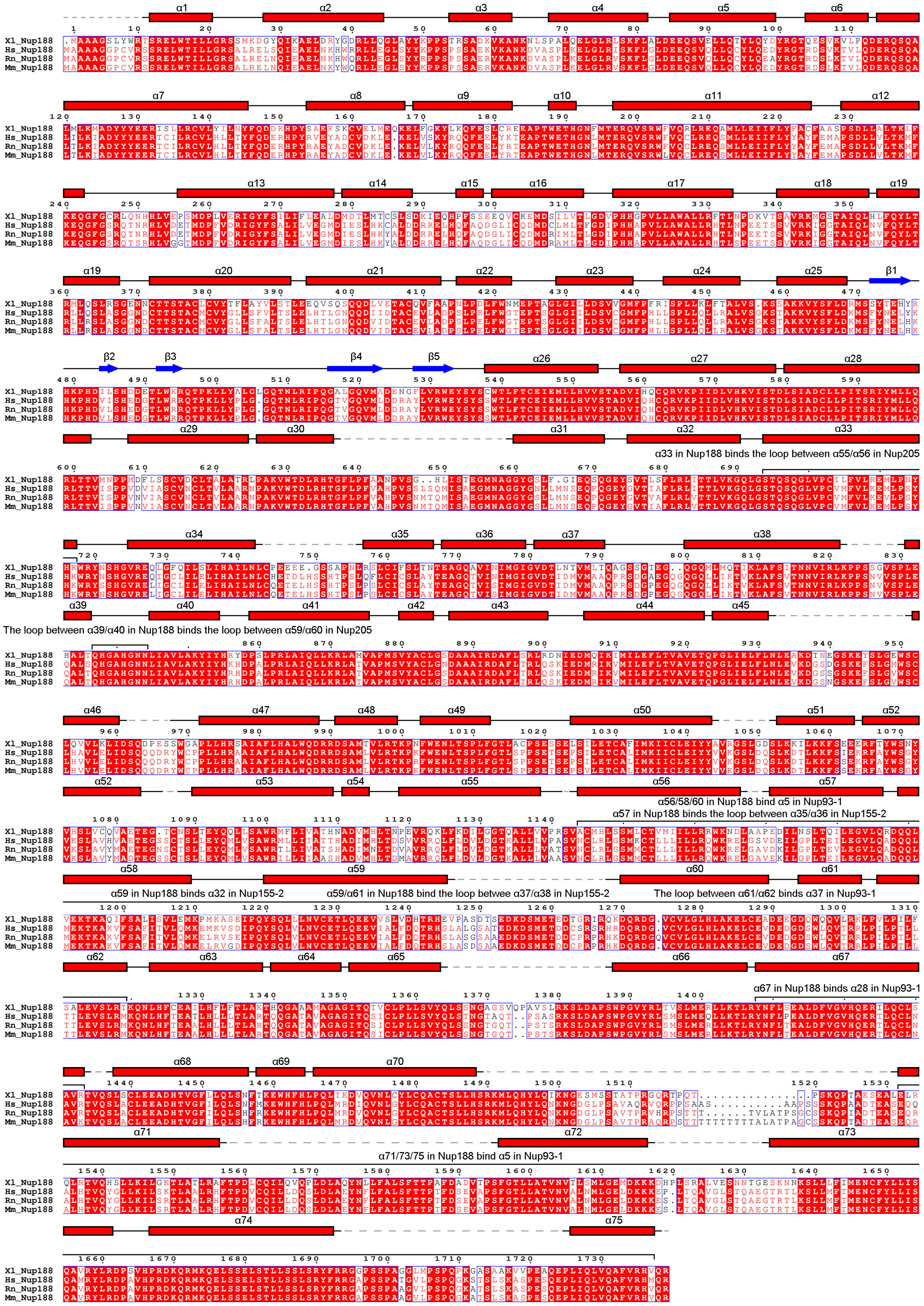

Supplementary information, Fig. S9 | Sequence alignment of Nup188 orthologues from *X. laevis* (Xl), *Homo sapiens* (Hs), *Rattus norvegicus* (Rn), and *Mus musculus* (Mm).

Shown here is the sequence alignment of the full-length Nup188 from indicated species. Conserved residues are boxed, with invariant ones shaded red. The secondary structural elements in Nup188 are indicated above the sequences. Structural elements interacting with other nucleoporins in the IR subunit are indicated.
