# Supplementary material for: Cryo-EM structure of the inner ring from the Xenopus laevis nuclear pore complex
Source: Cell Res. 2022 Mar 18;32(5):451–60. doi: 10.1038/s41422-022-00633-x (PMC9061766; doi:10.1038/s41422-022-00633-x)
Supplement: Supplementary file 10 — Supplementary information, Fig. S10 [file 41422_2022_633_MOESM10_ESM.pdf]

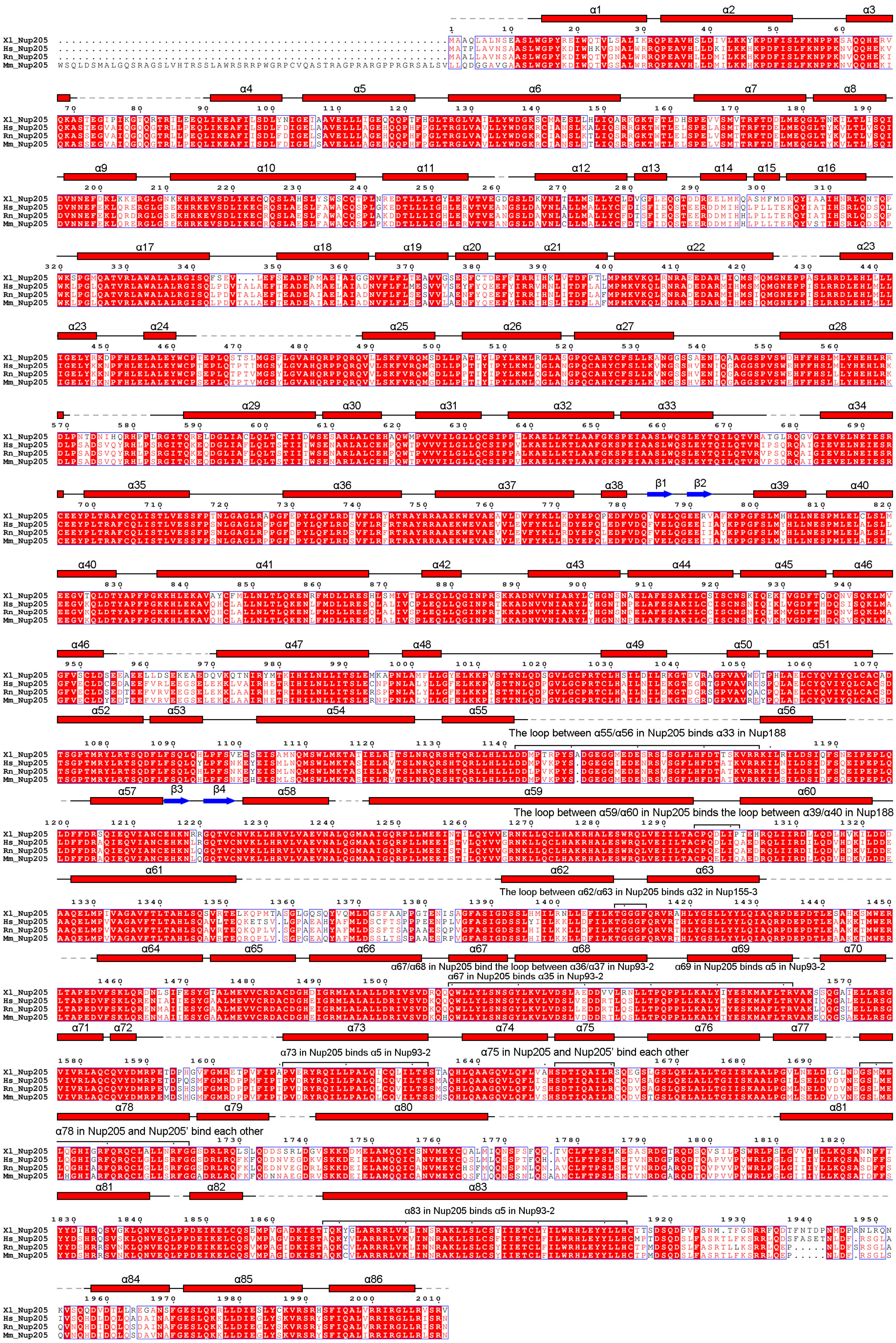

Supplementary information, Fig. S10 | Sequence alignment of Nup205 orthologues from *X. laevis* (Xl), *Homo sapiens* (Hs), *Rattus norvegicus* (Rn), and *Mus musculus* (Mm).

Shown here is the sequence alignment of the full-length Nup205 from indicated

species. Conserved residues are boxed, with invariant ones shaded red. The secondary structural elements in Nup205 are indicated above the sequences. Structural elements interacting with other nucleoporins in the IR subunit are indicated.
