# Supplementary material for: Cryo-EM structure of the inner ring from the Xenopus laevis nuclear pore complex
Source: Cell Res. 2022 Mar 18;32(5):451–60. doi: 10.1038/s41422-022-00633-x (PMC9061766; doi:10.1038/s41422-022-00633-x)
Supplement: Supplementary file 11 — Supplementary information, Fig. S11 [file 41422_2022_633_MOESM11_ESM.pdf]

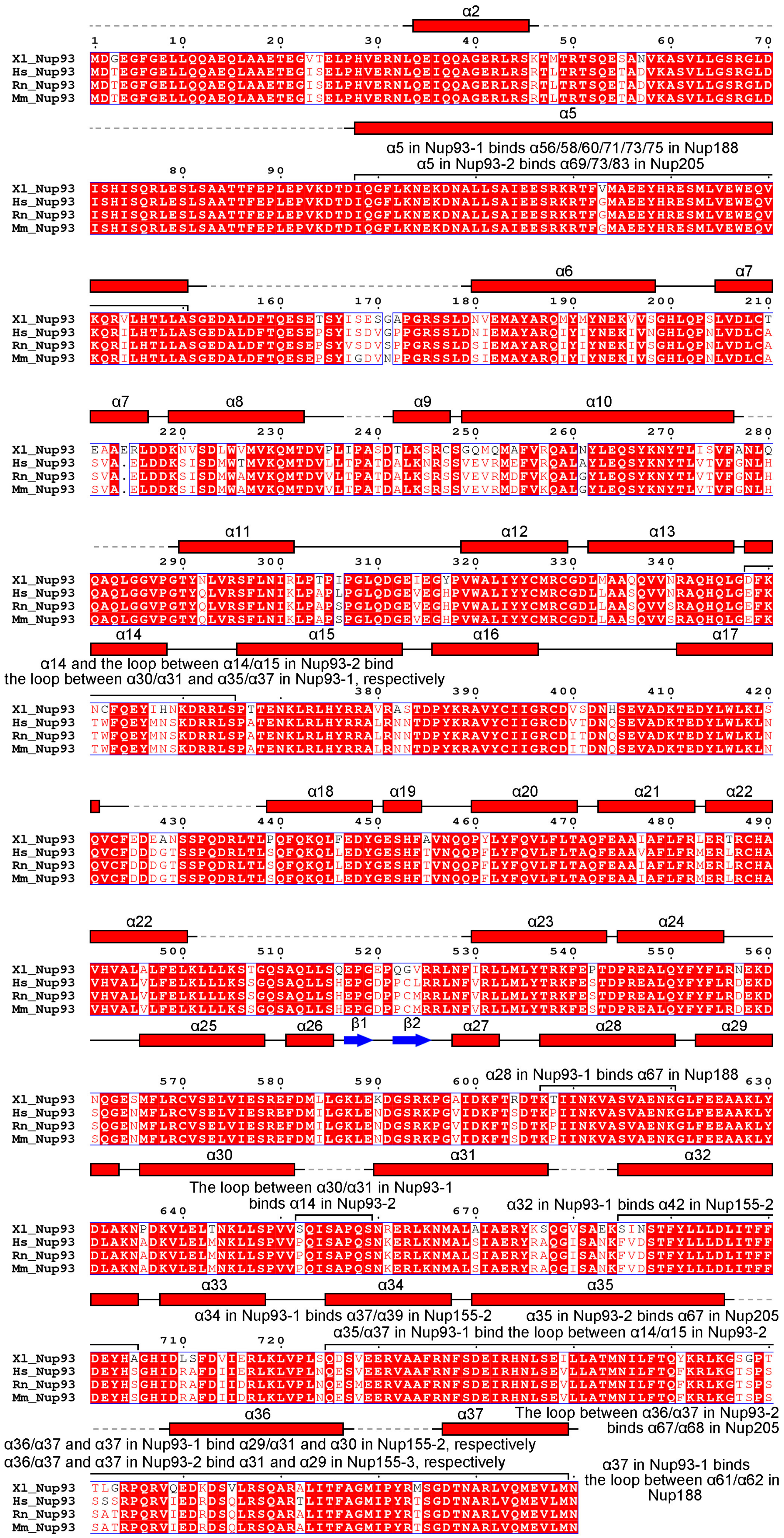
Xl_Nup93
Hs_Nup93
Rn_Nup93
Mm_Nup93
α5 in Nup93-1 binds α56/58/60/71/73/75 in Nup188
α5 in Nup93-2 binds α69/73/83 in Nup205
α14 and the loop between α14/α15 in Nup93-2 bind
the loop between α30/α31 and α35/α37 in Nup93-1, respectively
α28 in Nup93-1 binds α67 in Nup188
The loop between α30/α31 in Nup93-1
binds α14 in Nup93-2
α32 in Nup93-1 binds α42 in Nup155-2
α34 in Nup93-1 binds α37/α39 in Nup155-2
α35 in Nup93-2 binds α67 in Nup205
α35/α37 in Nup93-1 bind the loop between α14/α15 in Nup93-2
The loop between α36/α37 in Nup93-2
binds α67/α68 in Nup205
α36/α37 and α37 in Nup93-1 bind α29/α31 and α30 in Nup155-2, respectively
α36/α37 and α37 in Nup93-2 bind α31 and α29 in Nup155-3, respectively
α37 in Nup93-1 binds
the loop between α61/α62 in
Nup188

Supplementary information, Fig. S11 | Sequence alignment of Nup93 orthologues from *X. laevis* (Xl), *Homo sapiens* (Hs), *Rattus norvegicus* (Rn), and *Mus musculus* (Mm).

Shown here is the sequence alignment of the full-length Nup93 from indicated species. Conserved residues are boxed, with invariant ones shaded red. The secondary structural elements in Nup93-1 are indicated above the sequences. Structural elements interacting with other nucleoporins in the IR subunit are indicated.
