# Supplementary material for: Cryo-EM structure of the inner ring from the Xenopus laevis nuclear pore complex
Source: Cell Res. 2022 Mar 18;32(5):451–60. doi: 10.1038/s41422-022-00633-x (PMC9061766; doi:10.1038/s41422-022-00633-x)
Supplement: Supplementary file 12 — Supplementary information, Fig. S12 [file 41422_2022_633_MOESM12_ESM.pdf]

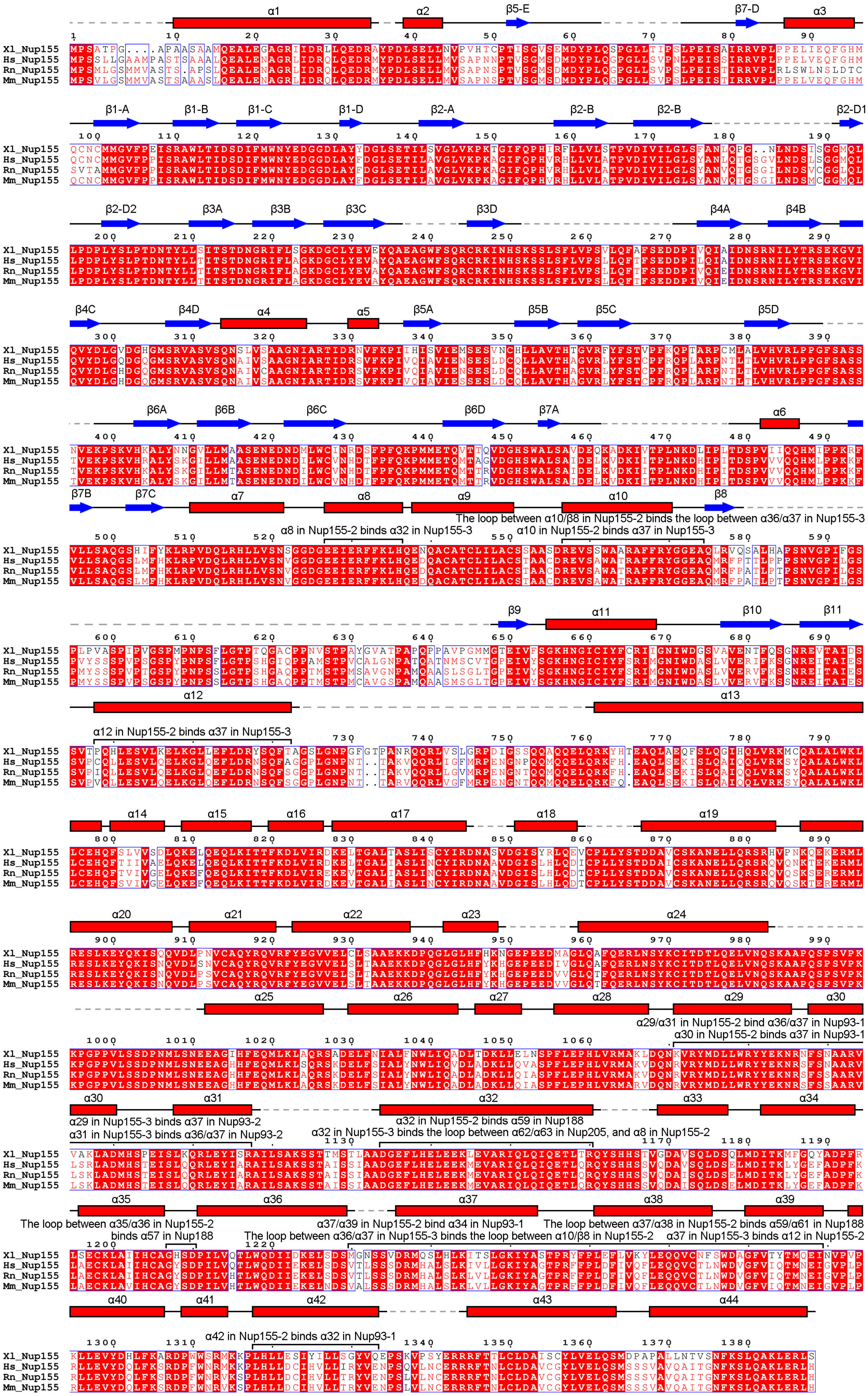
Xl_Nup155
Hs_Nup155
Rn_Nup155
Mm_Nup155
The loop between α10/β8 in Nup155-2 binds the loop between α36/α37 in Nup155-3
α8 in Nup155-2 binds α32 in Nup155-3
α10 in Nup155-2 binds α37 in Nup155-3
α12 in Nup155-2 binds α37 in Nup155-3
α29/α31 in Nup155-2 bind α36/α37 in Nup93-1
α30 in Nup155-2 binds α37 in Nup93-1
α29 in Nup155-3 binds α37 in Nup93-2
α31 in Nup155-3 binds α36/α37 in Nup93-2
α32 in Nup155-2 binds α59 in Nup188
α32 in Nup155-3 binds the loop between α62/α63 in Nup205, and α8 in Nup155-2
The loop between α35/α36 in Nup155-2 binds α57 in Nup188
α37/α39 in Nup155-2 bind α34 in Nup93-1
The loop between α37/α38 in Nup155-2 binds α59/α61 in Nup188
The loop between α36/α37 in Nup155-3 binds the loop between α10/β8 in Nup155-2
α37 in Nup155-3 binds α12 in Nup155-2
α42 in Nup155-2 binds α32 in Nup93-1

Supplementary information, Fig. S12 | Sequence alignment of Nup155 orthologues from *X. laevis* (Xl), *Homo sapiens* (Hs), *Rattus norvegicus* (Rn), and *Mus musculus* (Mm).

Shown here is the sequence alignment of the full-length Nup155 from indicated species. Conserved residues are boxed, with invariant ones shaded red. The secondary structural elements in Nup155-2 are indicated above the sequences. Structural elements interacting with other nucleoporins in the IR subunit are indicated.
