# Supplementary material for: Cryo-EM structure of the inner ring from the Xenopus laevis nuclear pore complex
Source: Cell Res. 2022 Mar 18;32(5):451–60. doi: 10.1038/s41422-022-00633-x (PMC9061766; doi:10.1038/s41422-022-00633-x)
Supplement: Supplementary file 13 — Supplementary information, Fig. S13 [file 41422_2022_633_MOESM13_ESM.pdf]

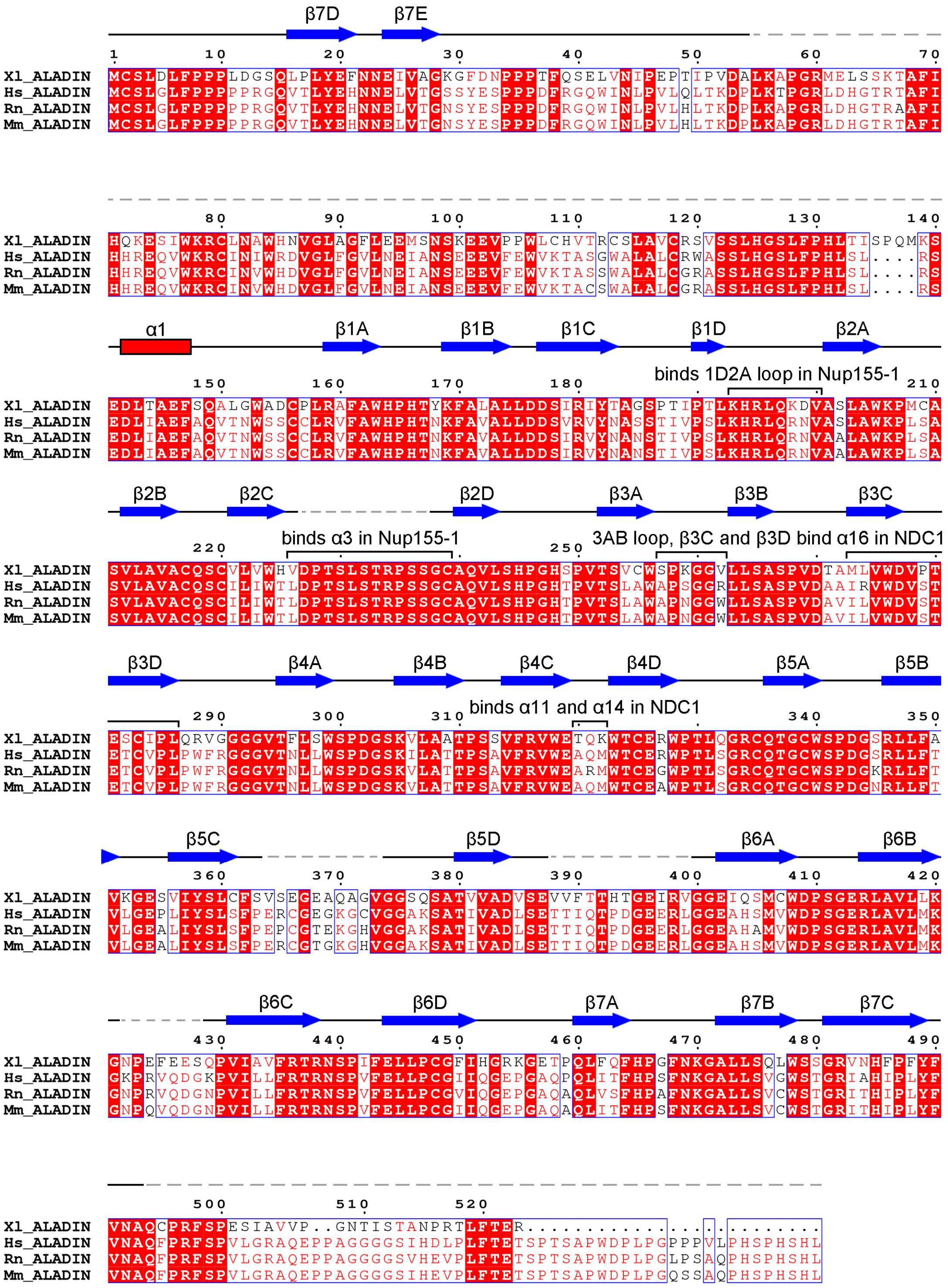

Supplementary information, Fig. S13 | Sequence alignment of ALADIN orthologues from *X. laevis* (Xl), *Homo sapiens* (Hs), *Rattus norvegicus* (Rn), and *Mus musculus* (Mm).

Shown here is the sequence alignment of the full-length ALADIN from indicated species. Conserved residues are boxed, with invariant ones shaded red. The secondary structural elements in ALADIN are indicated above the sequences. Structural elements interacting with other nucleoporins in the IR subunit are indicated.
