# Supplementary material for: Cryo-EM structure of the inner ring from the Xenopus laevis nuclear pore complex
Source: Cell Res. 2022 Mar 18;32(5):451–60. doi: 10.1038/s41422-022-00633-x (PMC9061766; doi:10.1038/s41422-022-00633-x)
Supplement: Supplementary file 14 — Supplementary information, Fig. S14 [file 41422_2022_633_MOESM14_ESM.pdf]

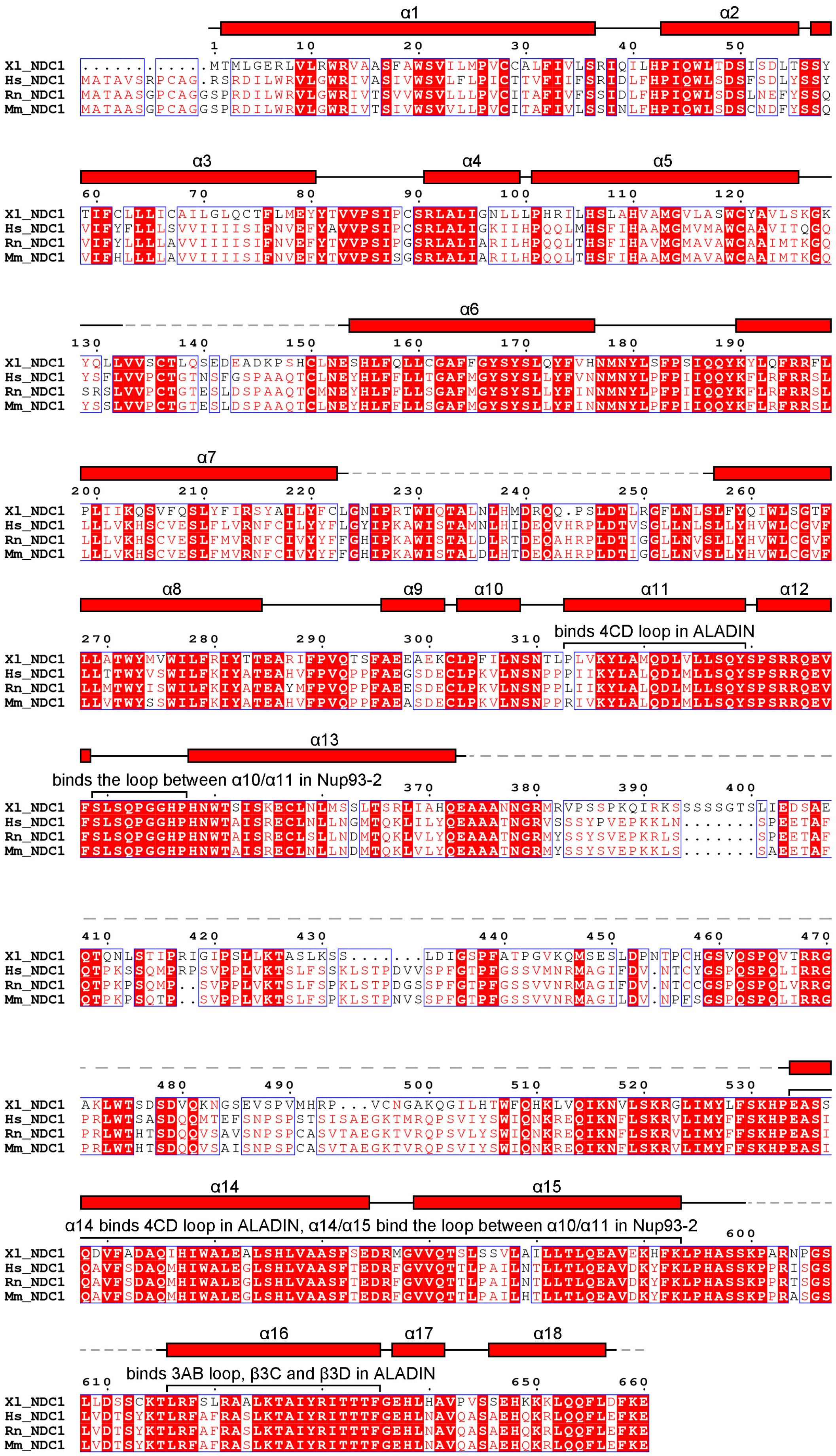
α1 α2
Xl_NDC1 ............MTMLGERLVLRWRVAASFAWSVILMPVCCALFIVLSRIQILHPIQWLTDSISDLTSSY
Hs_NDC1 MATAVSRPCAG.RSRDILWRVLGWRIVASIVWSVLFLPICTTVFIIFSRIDLFHPIQWLSDSFSDLYSSY
Rn_NDC1 MATAASGPCAGGSPRDILWRVLGWRIVTSVVWSVLLLPVCITAFIVFSSIDLFHPIQWLSDSLNEFYSSQ
Mm_NDC1 MATAASGPCAGGSPRDILWRVLGWRIVTSIVWSVVLLPVCITAFIVLSSINLFHPIQWLSDSCNDFYSSQ
α3 α4 α5
Xl_NDC1 TIFCLLLICAILGLQCTFLMEYYTVVPSIPCSRLALIGNLLLPHRILHSLAHVAMGVLASWCYAVLSKGK
Hs_NDC1 VIFYFLLLSVVIIIISIFNVEFYAVVPSIPCSRLALIGKIIHPQQLMHSFIHAAMGMVMAWCAAVITQGQ
Rn_NDC1 VIFYLLLLAVVIIIISIFNVEFYTVVPSIPGSRLALIARILHPQQLTHSFIHAVMGMAVAWCAAIMTKGQ
Mm_NDC1 VIFHLLLLAVVIIIISIFNVEFYTVVPSISGSRLALIARILHPQQLTHSFIHAAMGMAVAWCAAIMTKGQ
α6
Xl_NDC1 YQLLVVSCTLQSEDEADKPSHCLNESHLFQLLCGAFFGYSYSLQYFVHNMNYLSFPSIQQYKYLQFRRFL
Hs_NDC1 YSFLVVPCTGTNSFGSPAAQTCLNEYHLFFLLTGAFMGYSYSLLYFVNNMNYLPFPIIQQYKFLRFRRSL
Rn_NDC1 SRSLVVPCTGTESLDSPAAQTCMNEYHLFFLLSGAFMGYSYSLLYFINNMNYLPFPIIQQYKFLRFRRSL
Mm_NDC1 YSSLVVPCTGTESLDSPAAQTCLNEYHLFFLLSGAFMGYSYSLLYFINNMNYLPFPIIQQYKFLRFRRSL
α7
Xl_NDC1 PLIIKQSVFQSLYFIRSYAILYFCLGNIPRTWIQTALNLHMDRQQ.PSLDTLRGFLNLSLFYQIWLSGTF
Hs_NDC1 LLLVKHSCVESLFLVRNFCILYYFLGYIPKAWISTAMNLHIDEQVHRPLDTVSGLLNLSLLYHVWLCGVF
Rn_NDC1 LLLVKHSCVESLFMVRNFCIVYYFFGHIPKAWISTALDLRTDEQAHRPLDTIGGLLNVSLLYHVWLCGVF
Mm_NDC1 LLLVKHSCVESLFMVRNFCIVYYFFGHIPKAWISTALDLHTDEQAHRPLDTIGGLLNVSLLYHVWLCGVF
α8 α9 α10 α11 α12
binds 4CD loop in ALADIN
Xl_NDC1 LLATWYMVWILFRIYTTEARIFPVQTSFAEEAEKCLPFILNSNTLPLVKYLAMQDLVLLSQYSPSRRQEV
Hs_NDC1 LLTTWYVSWILFKIYATEAHVFPVQPPFAEGSDECLPKVLNSNPPPIIKYLALQDLMLLSQYSPSRRQEV
Rn_NDC1 LLMTWYISWILFKIYATEAYMFPVQPPFAEESDECLPKVLNSNPPLIIKYLALQDLMLLSQYSPSRRQEV
Mm_NDC1 LLVTWYSSWILFKIYATEAHVFPVQPPFAEASDECLPKVLNSNPPRIVKYLALQDLMLLSQYSPSRRQEV
α13
binds the loop between α10/α11 in Nup93-2
Xl_NDC1 FSLSQPGGHPHNWTSISKECLNLMSSLTSRLIAHQEAAANNGRMRVPSSPKQIRKSSSSSGTSLIEDSAE
Hs_NDC1 FSLSQPGGHPHNWTAISRECLNLLNGMTQKLILYQEAAATNGRVSSSYPVEPKKLN.......SPEETAF
Rn_NDC1 FSLSQPGGHPHNWTAISRECLSLLNDMTQKLVLYQEAAATNGRMYSSYSVEPKRLS.......SPEETAF
Mm_NDC1 FSLSQPGGHPHNWTAISRECLNLLNDMTQKLVLYQEAAATNGRMYSSYSVEPKKLS.......SAEETAF
Xl_NDC1 QTQNLSTIPRIGIPSLLKTASLKSS......LDIGSPFATPGVKQMSESLDPNTPCHGSVQSPQVTRRG
Hs_NDC1 QTPKSSQMPRPSVPPLVKTSLFSSKLSTPDVVSPFGTPFGSSVMNRMAGIFDV.NTCYGSPQSPQLIRRG
Rn_NDC1 QTPKPSQMP..SVPPLVKTSLFSPKLSTPDGSSPFGTPFGSSVVNRMAGIFDV.NTCCGSPQSPQLVRRG
Mm_NDC1 QTPKPSQTP..SVPPLVKTSLFSPKLSTPNVSSPFGTPFGSSVVNRMAGILDV.NPFSGSPQSPQLIRRG
Xl_NDC1 AKLWTSDSDVQKNGSEVSPVMHRP...VCNGAKQGILHTWFQHKLVQIKNVLSKRGLIMYLFSKHPEASS
Hs_NDC1 PRLWTSASDQQMTEFSNPSPSTSISAEGKTMRQPSVIYSWIQNKREQIKNFLSKRVLIMYFFSKHPEASI
Rn_NDC1 PRLWTHTSDQQVSAVSNPSPCASVTAEGKTVRQPSVLYSWIQNKREQIKNFLSKRVLIMYFFSKHPEASI
Mm_NDC1 PRLWTHTSDQQVSAISNPSPCASVTAEGKTVRQPSVIYSWIQNKREQIKNFLSKRVLIMYFFSKHPEASI
α14 α15
α14 binds 4CD loop in ALADIN, α14/α15 bind the loop between α10/α11 in Nup93-2
Xl_NDC1 QDVFADAQIHIWALEALSHLVAASFSEDRMGVVQTSLSSVLAILLTLQEAVEKHFKLPHASSKPARNPGS
Hs_NDC1 QAVFSDAQMHIWALEGLSHLVAASFTEDRFGVVQTTLPAILNTLLTLQEAVDKYFKLPHASSKPPRISGS
Rn_NDC1 QAVFSDAQMHIWALEGLSHLVAASFTEDRFGVVQTTLPAILNTLLTLQEAVDKYFKLPHASSKPPRTSGS
Mm_NDC1 QAVFSDAQMHIWALEGLSHLVAASFTEDRFGVVQTTLPAILHTLLTLQEAVDKYFKLPHASSKPPRASGS
α16 α17 α18
binds 3AB loop, β3C and β3D in ALADIN
Xl_NDC1 LLDSSCKTLRFSLRAALKTAIYRITTTFGEHLHAVPVSSEHKKKLQQFLDFKE
Hs_NDC1 LVDTSYKTLRFAFRASLKTAIYRITTTFGEHLNAVQASAEHQKRLQQFLEFKE
Rn_NDC1 LVDTSYKTLRFAFRASLKTAIYRITTTFGEHLNAVQASAEHQKRLQQFLEFKE
Mm_NDC1 LVDTSYKTLRFAFRASLKTAIYRITTTFGEHLNAVQASAEHQKRLQQFLEFKE

Supplementary information, Fig. S14 | Sequence alignment of NDC1 orthologues from *X. laevis* (Xl), *Homo sapiens* (Hs), *Rattus norvegicus* (Rn), and *Mus musculus* (Mm).

Shown here is the sequence alignment of the full-length NDC1 from indicated species. Conserved residues are boxed, with invariant ones shaded red. The secondary structural elements in NDC1 are indicated above the sequences. Structural elements interacting with other nucleoporins in the IR subunit are indicated.
