# Supplementary material for: Cryo-EM structure of the inner ring from the Xenopus laevis nuclear pore complex
Source: Cell Res. 2022 Mar 18;32(5):451–60. doi: 10.1038/s41422-022-00633-x (PMC9061766; doi:10.1038/s41422-022-00633-x)
Supplement: Supplementary file 15 — Supplementary information, Fig. S15 [file 41422_2022_633_MOESM15_ESM.pdf]

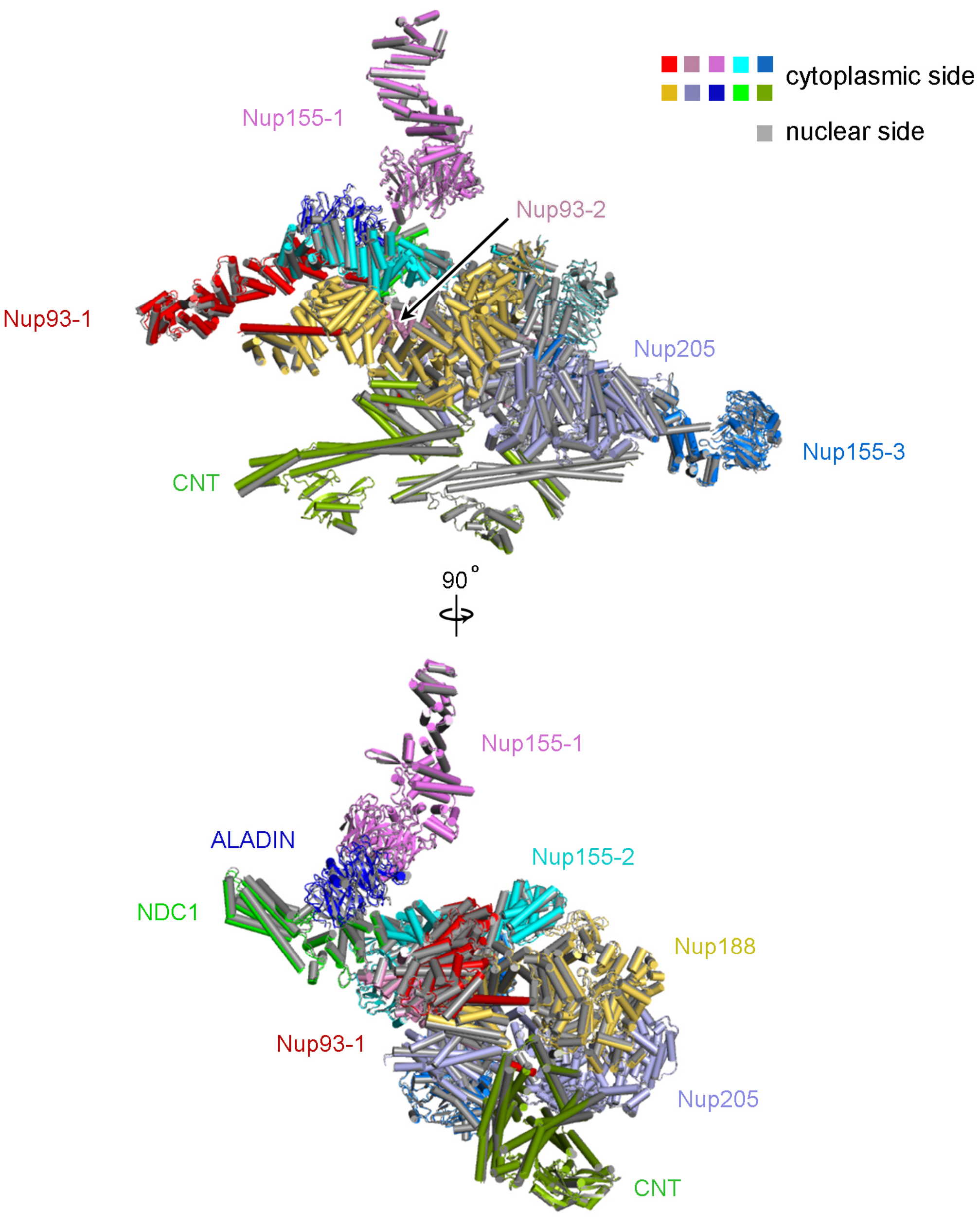

RMSD: 1.343 Å (8911 Cα aligned)

Supplementary information, Fig. S15 | The two halves of the IR subunit are nearly identical.

The two halves of the IR subunit can be superimposed with a root-mean-squared deviation (RMSD) of ~ 1.34 Å over 8,911 aligned Cα atoms. Two perpendicular views are shown.
