# Supplementary material for: Cryo-EM structure of the inner ring from the Xenopus laevis nuclear pore complex
Source: Cell Res. 2022 Mar 18;32(5):451–60. doi: 10.1038/s41422-022-00633-x (PMC9061766; doi:10.1038/s41422-022-00633-x)
Supplement: Supplementary file 16 — Supplementary information, Fig. S16 [file 41422_2022_633_MOESM16_ESM.pdf]

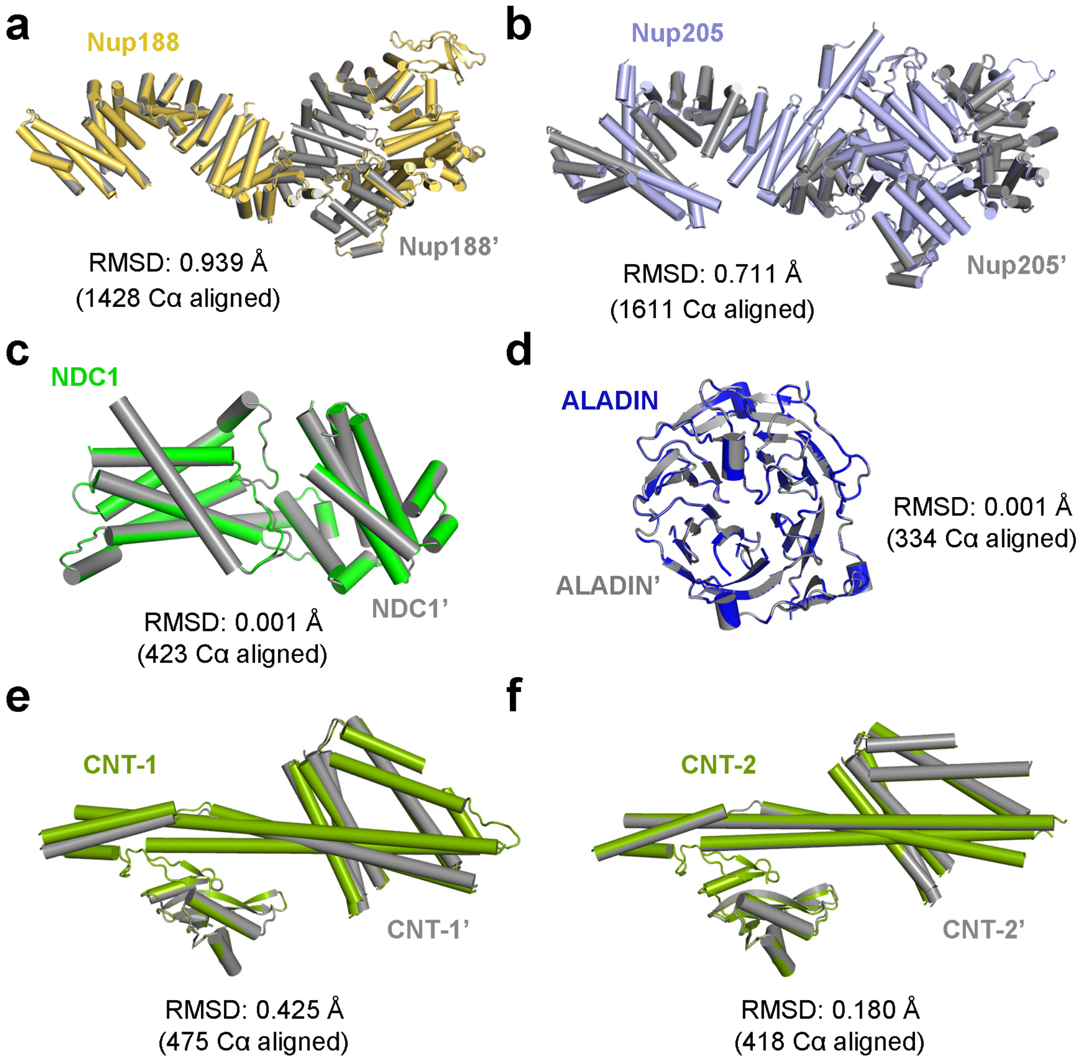

Supplementary information, Fig. S16 | Structural comparison of corresponding IR nucleoporins on the cytoplasmic and the nuclear sides.

Structures of the indicated components on the cytoplasmic side are color coded, and the corresponding ones on the nuclear side are colored grey and denoted with an apostrophe.
