# Supplementary material for: Cryo-EM structure of the inner ring from the Xenopus laevis nuclear pore complex
Source: Cell Res. 2022 Mar 18;32(5):451–60. doi: 10.1038/s41422-022-00633-x (PMC9061766; doi:10.1038/s41422-022-00633-x)
Supplement: Supplementary file 17 — Supplementary information, Fig. S17 [file 41422_2022_633_MOESM17_ESM.pdf]

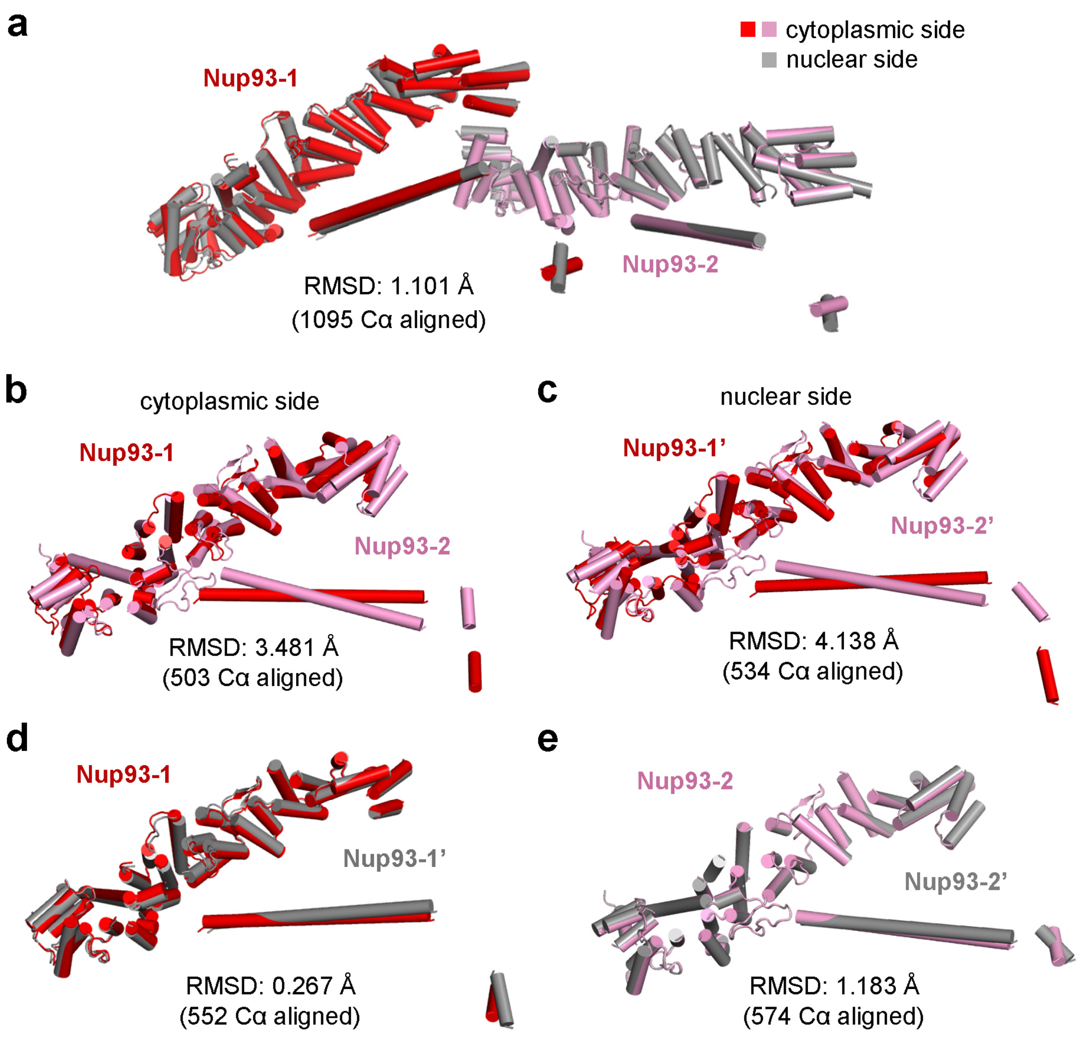

Supplementary information, Fig. S17 | Structural comparison of Nup93 molecules within the IR subunit.

Structures of the indicated components on the cytoplasmic side are color coded, and the corresponding ones on the nuclear side, except Nup93-1’ and Nup93-2’ in panel c, are colored grey and denoted with an apostrophe.
