# Supplementary material for: Cryo-EM structure of the inner ring from the Xenopus laevis nuclear pore complex
Source: Cell Res. 2022 Mar 18;32(5):451–60. doi: 10.1038/s41422-022-00633-x (PMC9061766; doi:10.1038/s41422-022-00633-x)
Supplement: Supplementary file 18 — Supplementary information, Fig. S18 [file 41422_2022_633_MOESM18_ESM.pdf]

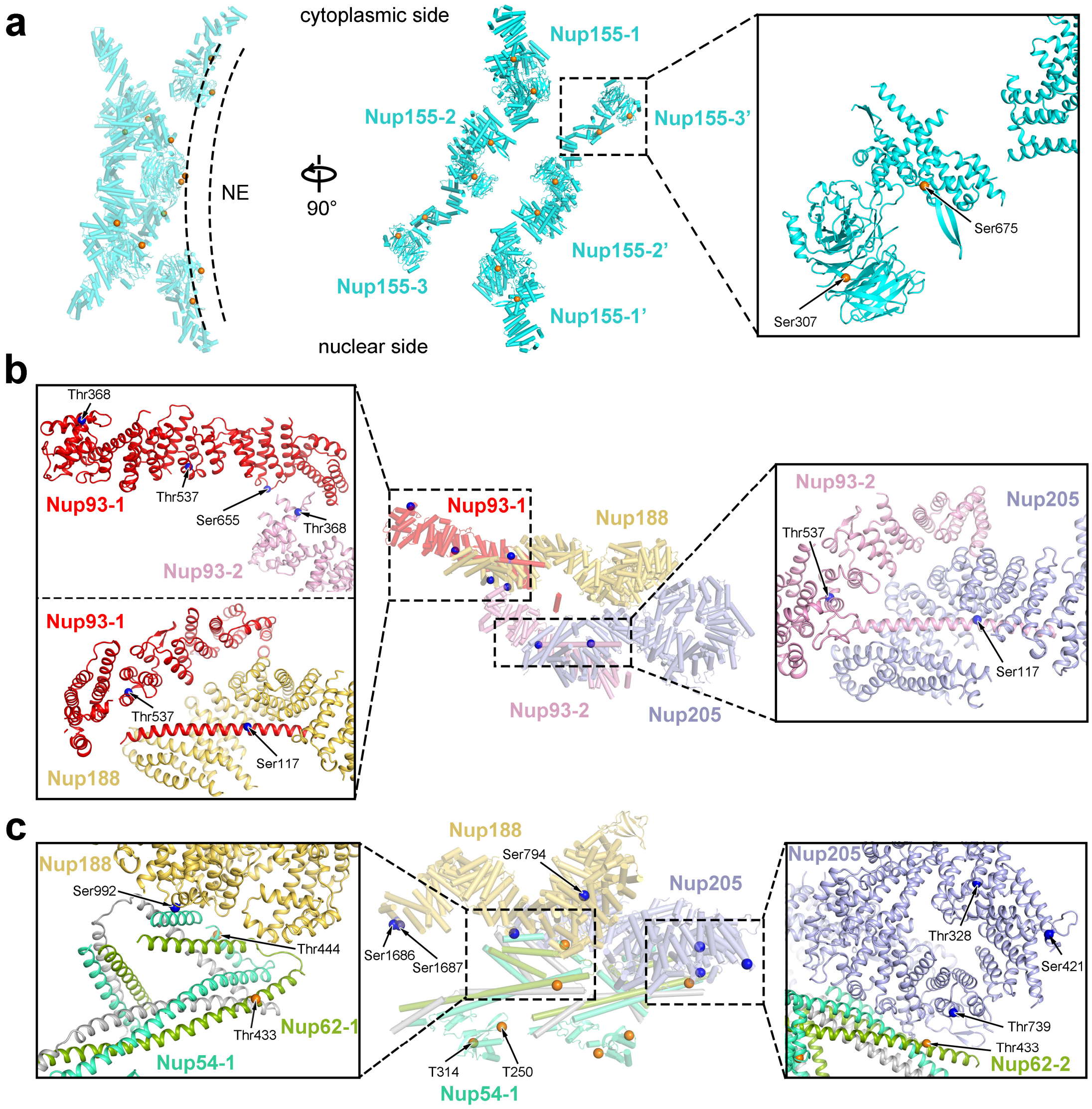

Supplementary information, Fig. S18 | Structural mapping of phosphorylation sites on select components of the IR subunit.

a, Mapping of the phosphorylation sites Ser307 and Ser675 onto Nup155. Based on our structure, both Ser307 and Ser675 face the NE. Phosphorylation may alter their interaction with the membrane, thus potentially affecting membrane localization of Nup155. **b**, Mapping of the phosphorylation sites Ser117, Thr368, Thr537, and Ser655 onto Nup93. Phosphorylation of Ser117 may affect the interaction between the extended helix α5 and Nup205 or Nup188. Phosphorylation of Thr368 or Ser655 may alter the interface between Nup93-1 and Nup93-2. Phosphorylation of Thr537 may change the local conformation. **c**, Mapping of phosphorylation sites onto Nup188, Nup205, and CNT. The phosphorylated residues include Ser794/Ser992/Ser1686/Ser1687 in Nup188, Thr328/Ser421/Thr739 in Nup205, Thr250/Thr314/Thr444 in Nup54, and Thr433 in Nup62. Their phosphorylation likely affects their mutual interactions or local conformations.
