# Supplementary material for: Cryo-EM structure of the inner ring from the Xenopus laevis nuclear pore complex
Source: Cell Res. 2022 Mar 18;32(5):451–60. doi: 10.1038/s41422-022-00633-x (PMC9061766; doi:10.1038/s41422-022-00633-x)
Supplement: Supplementary file 19 — Supplementary information, Fig. S19 [file 41422_2022_633_MOESM19_ESM.pdf]

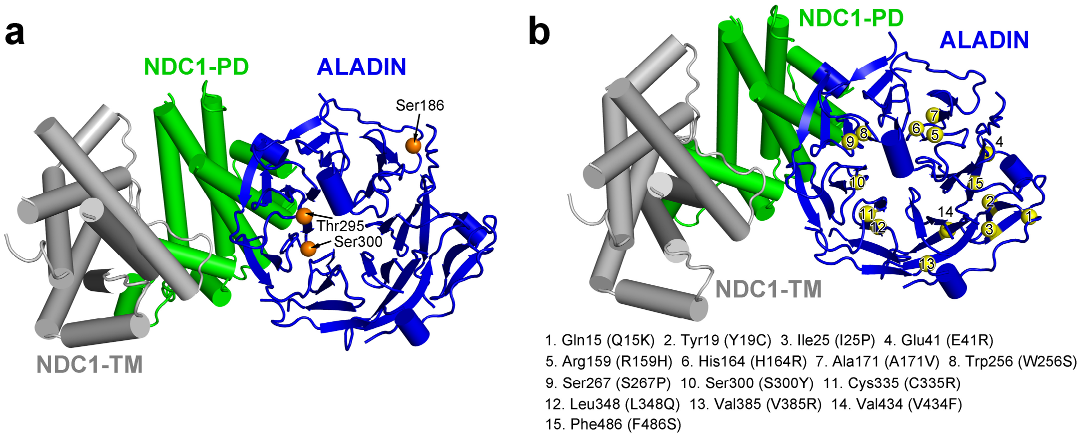

Supplementary information, Fig. S19 | Structural mapping of phosphorylation sites and disease mutation sites on ALADIN.

a, Mapping of three phosphorylation sites (Ser186, Thr295, and Ser300) on ALADIN. Phosphorylation of these sites may alter the local conformation of ALADIN. **b**, Mapping of 15 residues in ALADIN that are mutated in the triple A syndrome (AAAS)[1,2]. Each of these mutations is likely to affect the local conformation and/or stability of ALADIN.

References

1 Handschug, K. et al. Triple A syndrome is caused by mutations in AAAS, a new WD-repeat protein gene. Hum Mol Genet 10, 283-290, doi:10.1093/hmg/10.3.283 (2001).

2 Huebner, A. et al. The triple A syndrome is due to mutations in ALADIN, a novel member of the nuclear pore complex. Endocr Res 30, 891-899, doi:10.1081/erc-200044138 (2004).
