# Supplementary material for: Cryo-EM structure of the inner ring from the Xenopus laevis nuclear pore complex
Source: Cell Res. 2022 Mar 18;32(5):451–60. doi: 10.1038/s41422-022-00633-x (PMC9061766; doi:10.1038/s41422-022-00633-x)
Supplement: Supplementary file 20 — Supplementary information, Fig. S20 [file 41422_2022_633_MOESM20_ESM.pdf]

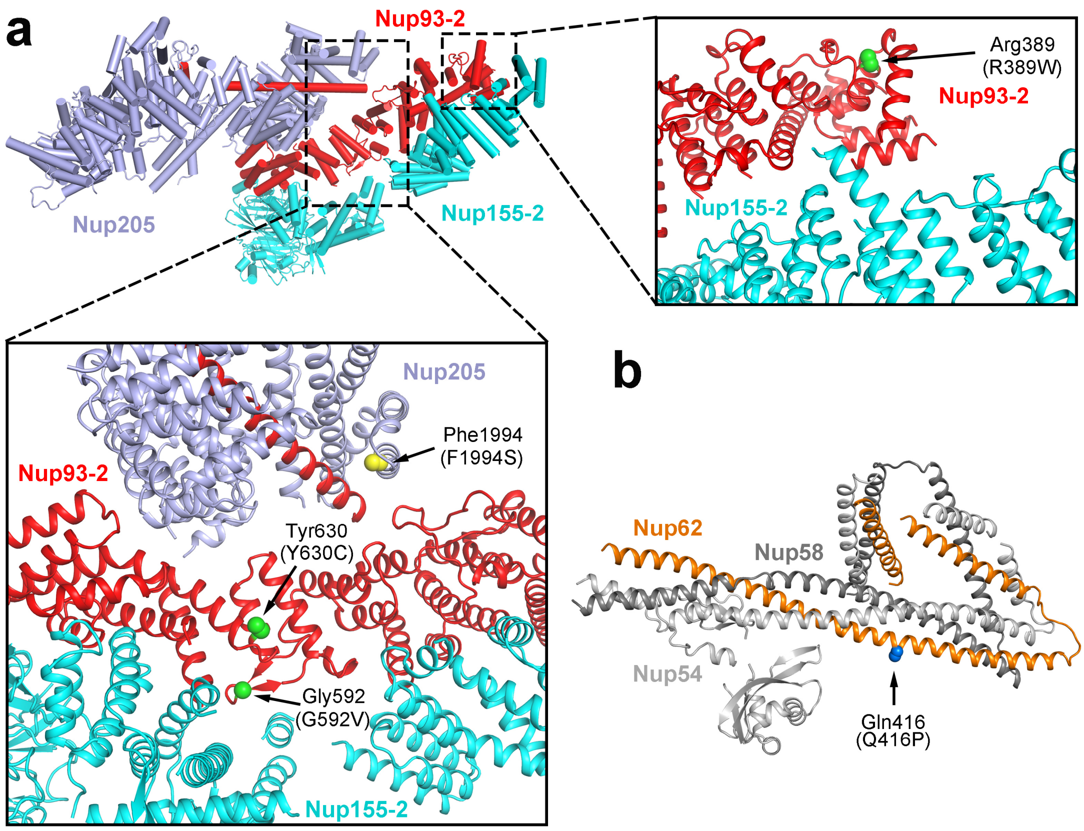

Supplementary information, Fig. S20 | Structural mapping of disease-derived mutations onto components of the IR subunit.

a, Mapping of four residues that are targeted for mutations in steroid-resistant nephrotic syndrome (SRNS)[1]. These four residues are Arg389/Gly592/Tyr630 in Nup93 and Phe1994 in Nup205. The mutations, R389W, Y630C, G592V and F1994S, may alter the local conformation. **b**, Mapping of the disease autosomal recessive infantile bilateral striatal necrosis (IBSN) mutation Q416P in Nup62[2]. The mutation of Gln416 to a helix-breaking residue Pro in the middle of an extended helix may destabilize the helical conformation.

References

1 Braun, D. A. et al. Mutations in nuclear pore genes NUP93, NUP205 and XPO5 cause steroid-resistant nephrotic syndrome. Nat Genet 48, 457-465, doi:10.1038/ng.3512 (2016).

2 Basel-Vanagaite, L. et al. Mutated nup62 causes autosomal recessive infantile bilateral striatal necrosis. Ann Neurol 60, 214-222, doi:10.1002/ana.20902 (2006).
