# Supplementary material for: Cryo-EM structure of the inner ring from the Xenopus laevis nuclear pore complex
Source: Cell Res. 2022 Mar 18;32(5):451–60. doi: 10.1038/s41422-022-00633-x (PMC9061766; doi:10.1038/s41422-022-00633-x)
Supplement: Supplementary file 21 — Supplementary information, Fig. S21 [file 41422_2022_633_MOESM21_ESM.pdf]

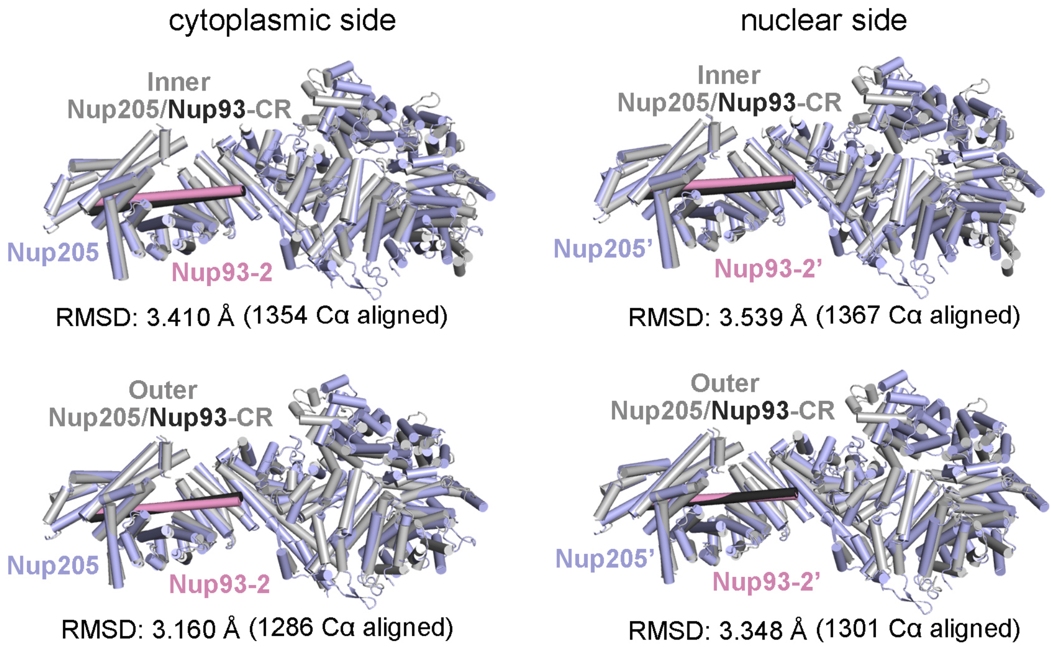

Supplementary information, Fig. S21 | A conserved interaction between Nup93 and Nup205 in the CR and the IR.

Structural comparison of the *X. laevis* Nup205/Nup93 pairs from the IR subunit with those from the CR subunit are shown. Structures of the Nup205/Nup93 pairs on the indicated positions, cytoplasmic side vs nuclear side or inner ring vs outer ring, are superimposed to the corresponding ones in the CR subunit.
