# Supplementary material for: Cryo-EM structure of the inner ring from the Xenopus laevis nuclear pore complex
Source: Cell Res. 2022 Mar 18;32(5):451–60. doi: 10.1038/s41422-022-00633-x (PMC9061766; doi:10.1038/s41422-022-00633-x)
Supplement: Supplementary file 22 — Supplementary information, Fig. S22 [file 41422_2022_633_MOESM22_ESM.pdf]

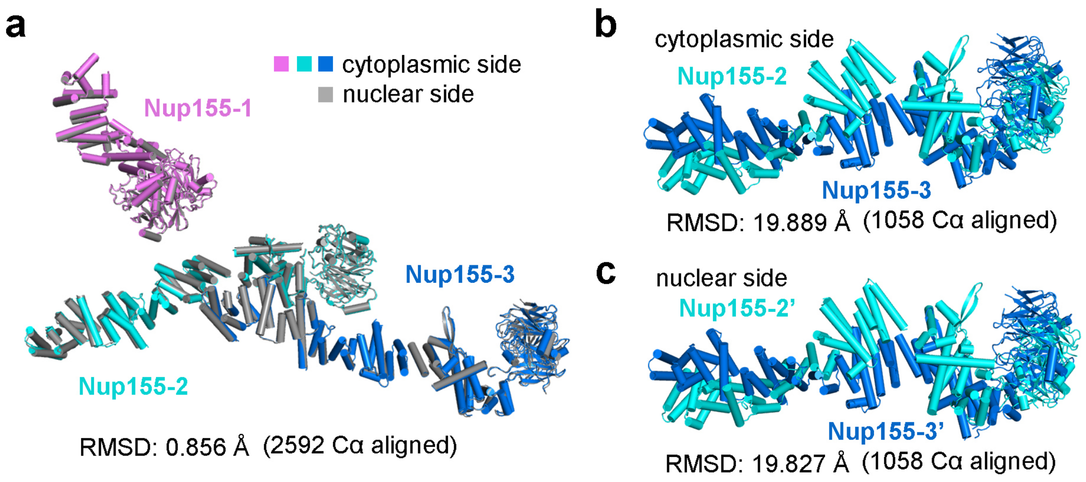

Supplementary information, Fig. S22 | Structural comparison of six Nup155 molecules from the IR subunit.

a, Structures of the three Nup155 molecules on the cytoplasmic side are nearly identical with those on the nuclear side. **b**, Nup155-2 and Nup155-3 on the cytoplasmic side show distinct conformations. **c**, Structural differences between Nup155-2' and Nup155-3'. Similar to the comparison between Nup155-2 and Nup155-3, their counterparts on the nuclear side also display different local conformations.
