# Supplementary material for: Cryo-EM structure of the inner ring from the Xenopus laevis nuclear pore complex
Source: Cell Res. 2022 Mar 18;32(5):451–60. doi: 10.1038/s41422-022-00633-x (PMC9061766; doi:10.1038/s41422-022-00633-x)
Supplement: Supplementary file 23 — Supplementary information, Fig. S23 [file 41422_2022_633_MOESM23_ESM.pdf]

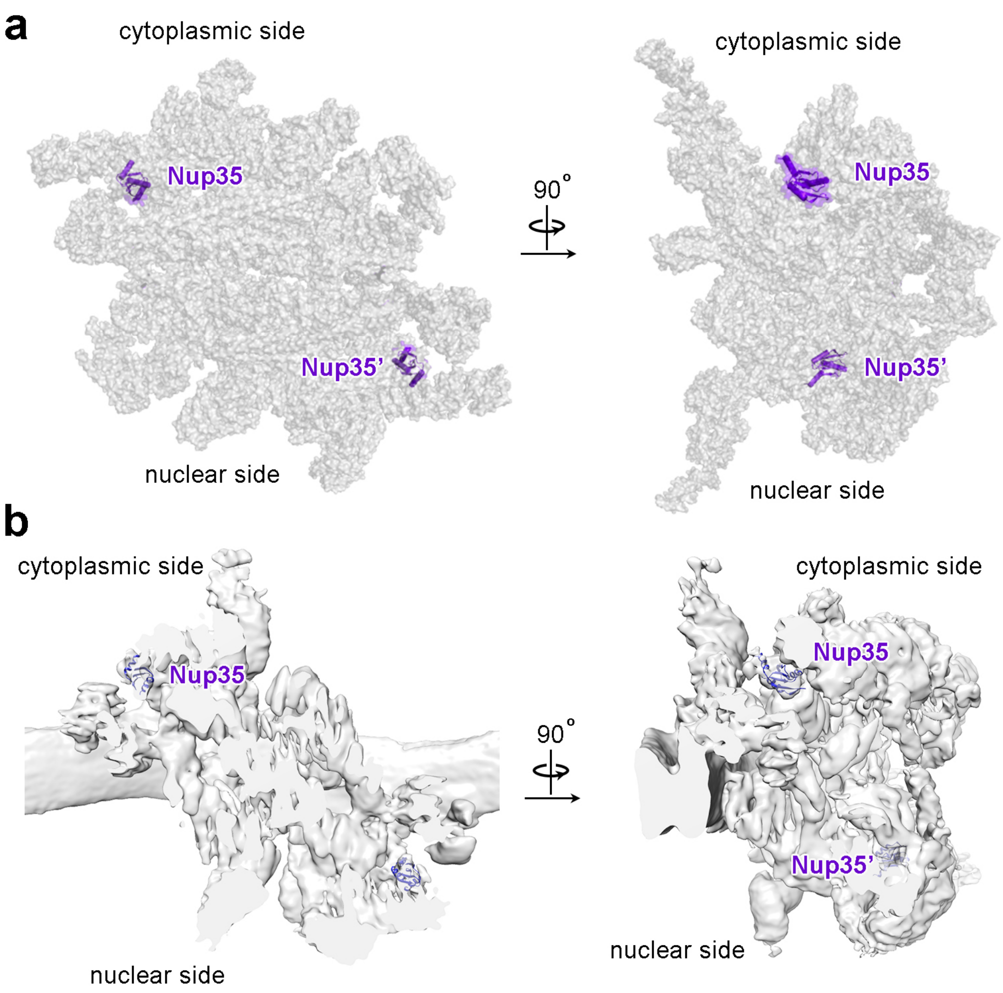


**Supplementary information, Fig. S23 | Potential locations of the RNA recognition motif (RRM) from Nup35.**

**a**, Two potential locations for the RRM domains of Nup35 are indicated in the structural model of the IR subunit. The IR subunit is shown in transparent surface representation. **b**, Two potential locations for the RRM domains of Nup35 are indicated in the EM map of the IR subunit.
