# Supplementary material for: Cryo-EM structure of the inner ring from the Xenopus laevis nuclear pore complex
Source: Cell Res. 2022 Mar 18;32(5):451–60. doi: 10.1038/s41422-022-00633-x (PMC9061766; doi:10.1038/s41422-022-00633-x)
Supplement: Supplementary file 24 — Supplementary information, Table S1 [file 41422_2022_633_MOESM24_ESM.pdf]

Supplementary information, Table S1 | Statistics of cryo-EM data collection and analysis.

**Data collection**				
EM equipment	Titan Krios (Thremo Fisher Scientific)			
Voltage (kV)	300			
Detector	Gatan K3			
Energy filter	Gatan GIF Quantum, 20 eV slit			
Pixel size (Å)	1.387			
Nominal Magnification	64,000			
Data set	Tilt0	Tilt30	Tilt45	Tilt55
Exposure rate ($e^-/(s·Å^2)$)	19.5	19.5	19.5	19.5
Number of frames	32	37	46	56
Total Electron exposure ($e^-/Å^2$)	50	57	87	100
Defocus range (µm)	-1.0~-4.0			
Total Number of images	10,040	7,557	14,107	14,439
Selected Number of images	8,145	6,171	10,319	9,112
Software	AutoEMation2			
**Reconstruction**				
Software	RELION3.0-beta/cryoSparc			
Number of used Particles	375,454	439,025	731,697	547,455
Symmetry/Final resolution (Å)	C1/6.0			
Masked regions	IR subunit core			
Number of Micrographs used	33,747			
Number of extracted particles	5,223,773			
Final number of particles	2,093,631			
Unmasked Resolution (Å) (0.5/0.143)	7.5/5.5			
Masked Resolution (Å) (0.5/0.143)	4.6/4.1			
Local Resolution Ranges (Å)	50-3.8			
Resolution Range due to anisotropy (Å)	4.4-4.1			
Final Resolution (Å)	4.2			
Map sharpening B-factor ($Å^2$)	-150			
Accuracy of rotation (˚)	0.99			
Accuracy of translation (pixels)	1.35			
EMDB number	EMD-32566			
**Model building**				
Software	Coot/Chimera			
Refinement software	Phenix			
PDB code	7WKK(IR subunit)			
Validation				
R.m.s deviations				
Bonds length (Å)	0.002			
Bonds Angle (˚)	0.360			
Ramachandran plot statistics (%)				
Preferred	98.00%			
Allowed	1.92%			
Outlier	0.08%			
