# Supplementary material for: Cryo-EM structure of the inner ring from the Xenopus laevis nuclear pore complex
Source: Cell Res. 2022 Mar 18;32(5):451–60. doi: 10.1038/s41422-022-00633-x (PMC9061766; doi:10.1038/s41422-022-00633-x)
Supplement: Supplementary file 25 — Supplementary information, Table S2 [file 41422_2022_633_MOESM25_ESM.pdf]

Supplementary information, Table S2 | Summary of model building for the IR subunit of the *X. laevis* NPC.

	Molecule **Vertebrates**/ Yeast	Copy No.	Length *Xenopus laevis*	UniProt No. *Xenopus laevis*	PDB code	Modeling	Model length	Resolution (Å)	Chain ID CR/NR side
**IR subunit**	**Nup205**/Nup192	2	2011	Q642R6	7FIK	RD	~1700	4.0~5.5	A/a
	**Nup188**/Nup188	2	1739	F6WXT2	AF/4KF7/4KF8	HM/RD	~1500	4.0~5.5	B/b
	**Nup93**/Nic96	4	820	Q7ZX96	7FIK	RD	~630	4.5~5.5	C/E/c/e
	**Nup155**/Nup155	6	1388	F6UHT0	AF	RD	~1150	4.5~6.0	D/F/M/d/f/m
	**Nup62**/Nsp1	4	552	Q6DIE3	AF/5C3L	RD	~170	5.0~6.5	H/L/h/l
	**Nup58**/Nup49	4	599	Q5EAX5	AF/5C3L	RD	~170	5.0~6.5	I/K/i/k
	**Nup54**/Nup57	4	535	K9ZTJ6	AF/5C3L	RD	~320	5.0~6.5	G/J/g/j
	**NDC1**/NDC1	2	660	Q6AX31	AF	RD	~430	5.5~6.5	N/n
	**ALADIN**/-	2	523	Q6DCM0	AF	RD	~370	5.5~6.5	O/o

Under the column labeled "Molecule", corresponding proteins from vertebrates and yeasts are listed, with vertebrate components in bold. Under the column labeled "PDB code", AF stands for AlphaFold (model generated from AlphaFold prediction). Under the column labeled "Modeling", HM stands for homology modeling, and RD stands for rigid docking and manual adjustment.
