# Supplementary material for: Cryo-EM structure of the inner ring from the Xenopus laevis nuclear pore complex
Source: Cell Res. 2022 Mar 18;32(5):451–60. doi: 10.1038/s41422-022-00633-x (PMC9061766; doi:10.1038/s41422-022-00633-x)
Supplement: Supplementary file 26 — Supplementary information, Table S3 [file 41422_2022_633_MOESM26_ESM.pdf]

Supplementary information, Table S3 | Summary of secondary structural elements in the structurally resolved nucleoporins of the IR subunit from the *X. laevis* NPC.

Protein	Full length (aa)	Residues modeled		Number of α-helices		Number of β-strands	
		cytoplasmic side	nuclear side	cytoplasmic side	nuclear side	cytoplasmic side	nuclear side
Nup205	2011	1709	1690	86	86	4	4
Nup188	1739	1502	1502	75	75	5	5
Nup93-1	820	641	641	32	32	2	2
Nup93-2		633	634	32	32	2	2
Nup155-1	1388	880	880	28	28	34	34
Nup155-2		1129	1138	44	44	34	34
Nup155-3		1111	1111	44	44	34	34
Nup62-1	552	166	163	3	3	0	0
Nup62-2		159	159	3	3	0	0
Nup58-1	599	171	171	4	4	0	0
Nup58-2		166	166	4	4	0	0
Nup54-1	535	219	198	6	6	2	2
Nup54-2		218	198	6	6	2	2
NDC1	660	431	431	18	18	0	0
ALADIN	523	367	367	1	1	29	29
